# Supplementary material for: DELVE: feature selection for preserving biological trajectories in single-cell data
Source: Nat Commun. 2024 Mar 29;15:2765. doi: 10.1038/s41467-024-46773-z (PMC10980758; doi:10.1038/s41467-024-46773-z)
Supplement: Supplementary file 1 — Supplementary Information [file 41467_2024_46773_MOESM1_ESM.pdf]

Supplementary Tables

Supplementary Table S1: Implemented feature selection method parameters

Method name	Supervision	Approach	Parameter description	Parameters	End Application
Random Forest classifier	supervised	supervised	cell type labels<br>number of trees	cell type labels<br>$\mathrm{n\_estimators}$ = 10, 100, 500	classification
DELVE	unsupervised	cell similarity graph<br>(dynamic features)	number of nearest neighbors<br>number of representative neighborhoods<br>number of modules<br>kernel bandwidth parameter	$k = 10$<br>$m = 1000$<br>$c = 3, 5, 10$<br>σ_i = 3rd-nearest neighbor distance	feature selection
Hotspot	unsupervised	cell similarity graph<br>(all features)	number of nearest neighbors<br>kernel bandwidth parameter<br>number of principal components<br>model	$k = 10$<br>σ_i = $k/3$-nearest neighbor distance<br>$\mathrm{n\_pcs} = 50$<br>danb, normal, none	module detection
Laplacian score	unsupervised	cell similarity graph<br>(all features)	number of nearest neighbors<br>kernel bandwidth parameter	$k = 10$<br>σ_i = 3rd-nearest neighbor distance	feature selection
Neighborhood variance	unsupervised	cell similarity graph<br>(all features)	-	-	feature selection
MCFS	unsupervised	cell similarity graph<br>(all features)	number of nearest neighbors<br>kernel bandwidth parameter<br>number of nonzero coefficients<br>number of eigenvectors	$k = 10$<br>σ_i = 3rd-nearest neighbor distance<br>p<br>c = number of known cell types	feature selection
SCMER	unsupervised	cell similarity graph<br>(all features)	number of nearest neighbors<br>number of principal components<br>lasso regression parameter<br>ridge regression parameter	$k = 10$<br>$\mathrm{n\_pcs} = 50$<br>lasso $= 3.87e - 4$<br>ridge $= 0$	feature selection
Max variance	unsupervised	variance-based	-	-	feature selection
Highly variable genes	unsupervised	variance-based	bins	20	feature selection
All features	-	-	-	-	-
Random features	-	-	-	-	-

Supplementary Figures

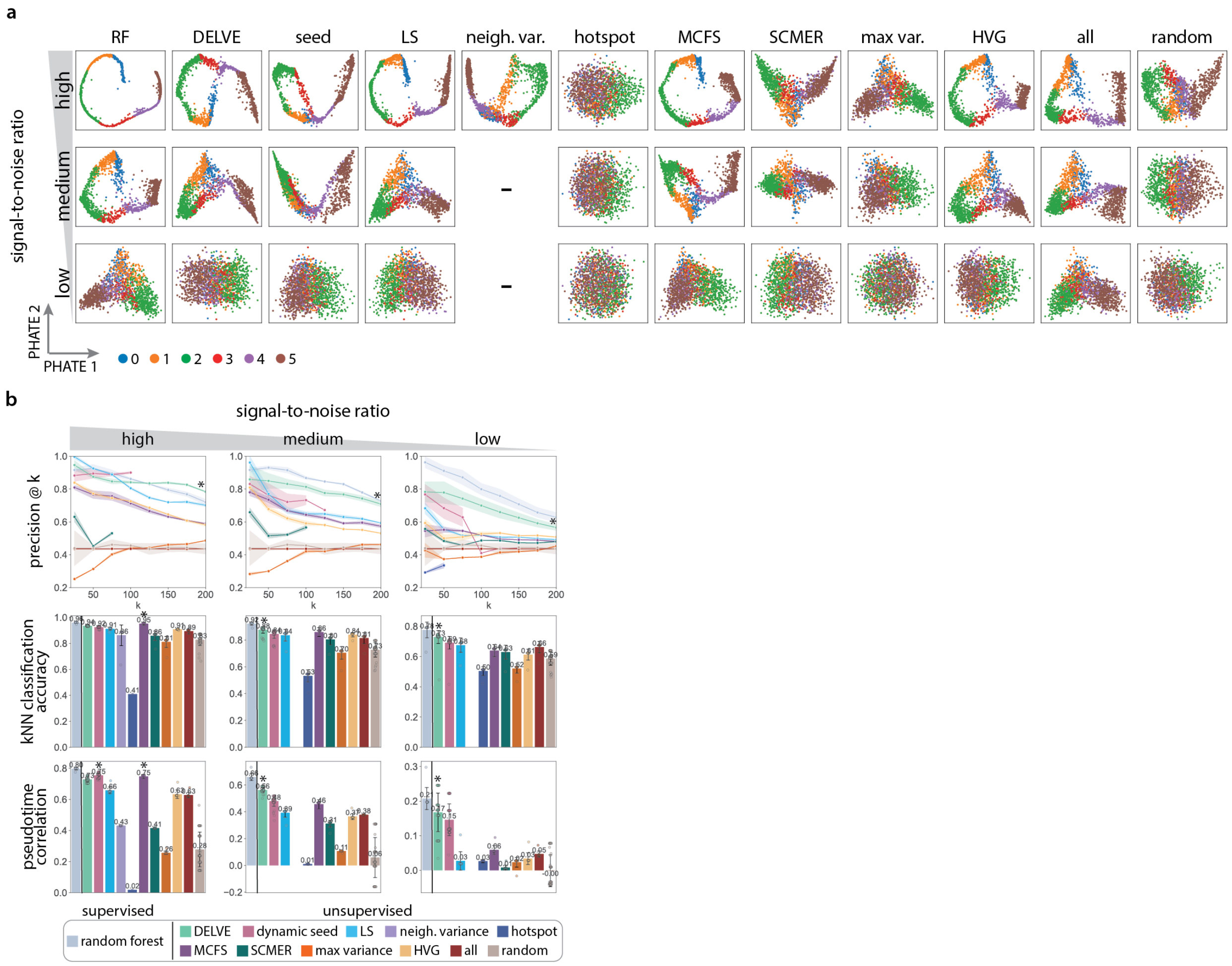

Supplementary Figure 1: Comparison of feature selection methods on preserving linear differentiation trajectories under a reduction in the signal-to-noise ratio. Ten single-cell RNA sequencing datasets with linear trajectories (1500 cells $\times$ 500 genes) were simulated with Splatter. Feature selection performance was subsequently assessed using the top $p = 100$ ranked genes for each feature selection strategy. (a) Example PHATE visualizations of simulated linear differentiation trajectories for twelve feature selection strategies when subjected to a reduction in the signal-to-noise ratio (high, medium, low). The signal-to-noise ratio was altered by modifying the biological coefficient of variation parameter within Splatter (high: $\texttt{BCV} = 0.1$, medium: $\texttt{BCV} = 0.25$, low: $\texttt{BCV} = 0.5$). This scaling factor controls the mean-variance relationship between genes, where lowly expressed genes are more variable than highly expressed genes. (b) Performance of twelve different feature selection methods when subjected to a reduction in the signal-to-noise ratio. Following feature selection (p = 100), trajectory preservation was quantitatively assessed according to several metrics: the precision of differentially expressed genes at k selected genes (top), k-NN classification accuracy (middle), and pseudotime correlation (bottom) across 10 random trials. Error bands represent the standard deviation over $n = 10$ simulation datasets. Barplots show the mean $\pm$ the standard deviation over $n = 10$ simulation datasets. * indicates the method with the highest median score. - indicates that the method identified no features. Source data are provided in a Source Data file.

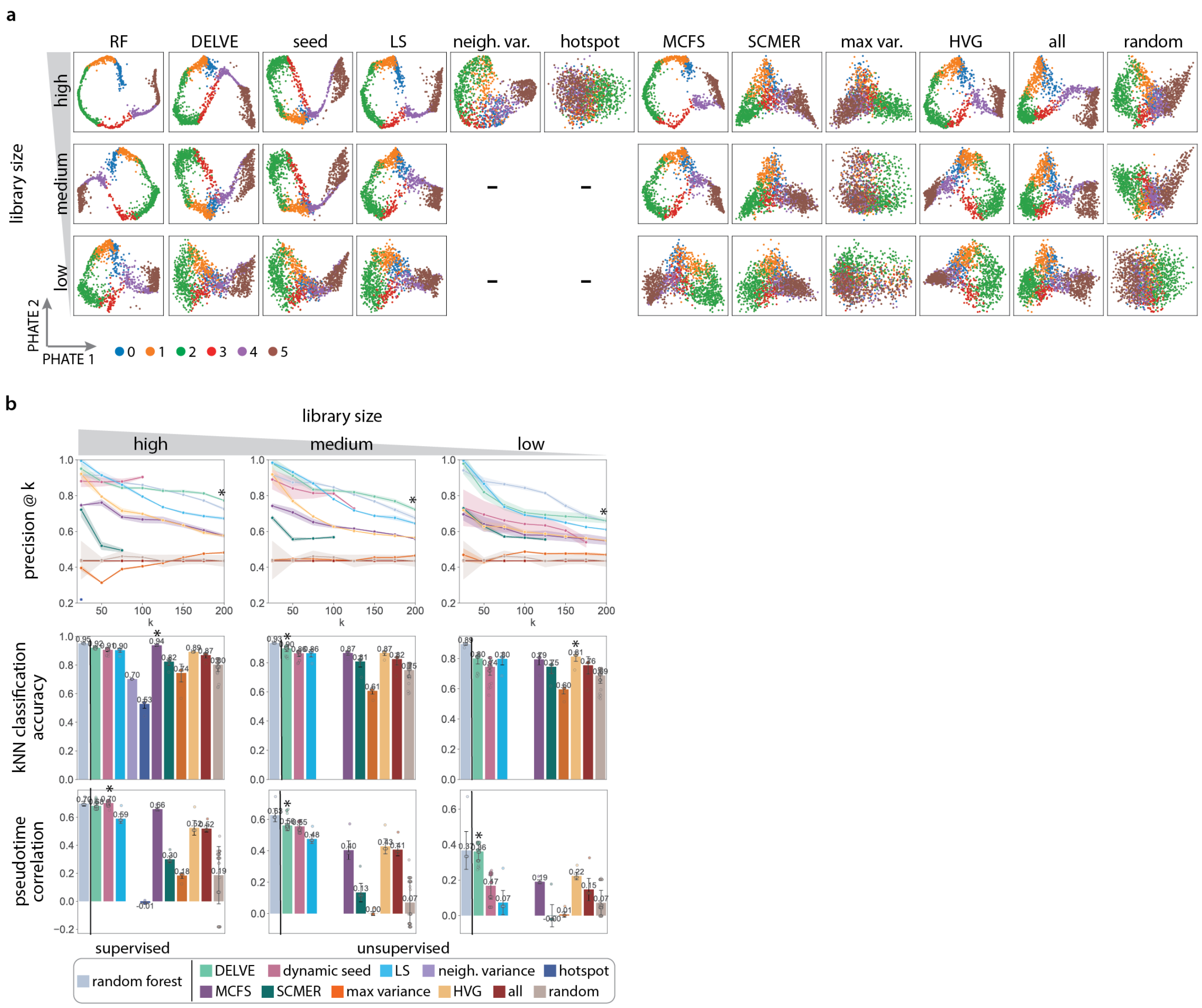

Supplementary Figure 2: Comparison of feature selection methods on preserving linear differentiation trajectories in the presence of library size noise. Ten single-cell RNA sequencing datasets with linear trajectories (1500 cells $\times$ 500 genes) were simulated with Splatter. Feature selection performance was subsequently assessed using the top $p = 100$ ranked genes for each feature selection strategy. (a) Example PHATE visualizations of simulated linear differentiation trajectories for twelve feature selection strategies when subjected to a reduction in the total mRNA count (high, medium, low). Library size was reduced by modifying the location parameter in the log-normal distribution in Splatter that specifies library size scaling factors (high: location = 12, medium: location = 11, low: location = 10). (b) Performance of twelve different feature selection methods when subjected to a reduction in total mRNA count. Following feature selection ($p = 100$), trajectory preservation was quantitatively assessed according to several metrics: the precision of differentially expressed genes at k selected genes (top), k-NN classification accuracy (middle), and pseudotime correlation (bottom) across 10 random trials. Error bands represent the standard deviation over $n = 10$ simulation datasets. Barplots show the mean $\pm$ the standard deviation over $n = 10$ simulation datasets. * indicates the method with the highest median score. - indicates that the method identified no features. Source data are provided in a Source Data file.

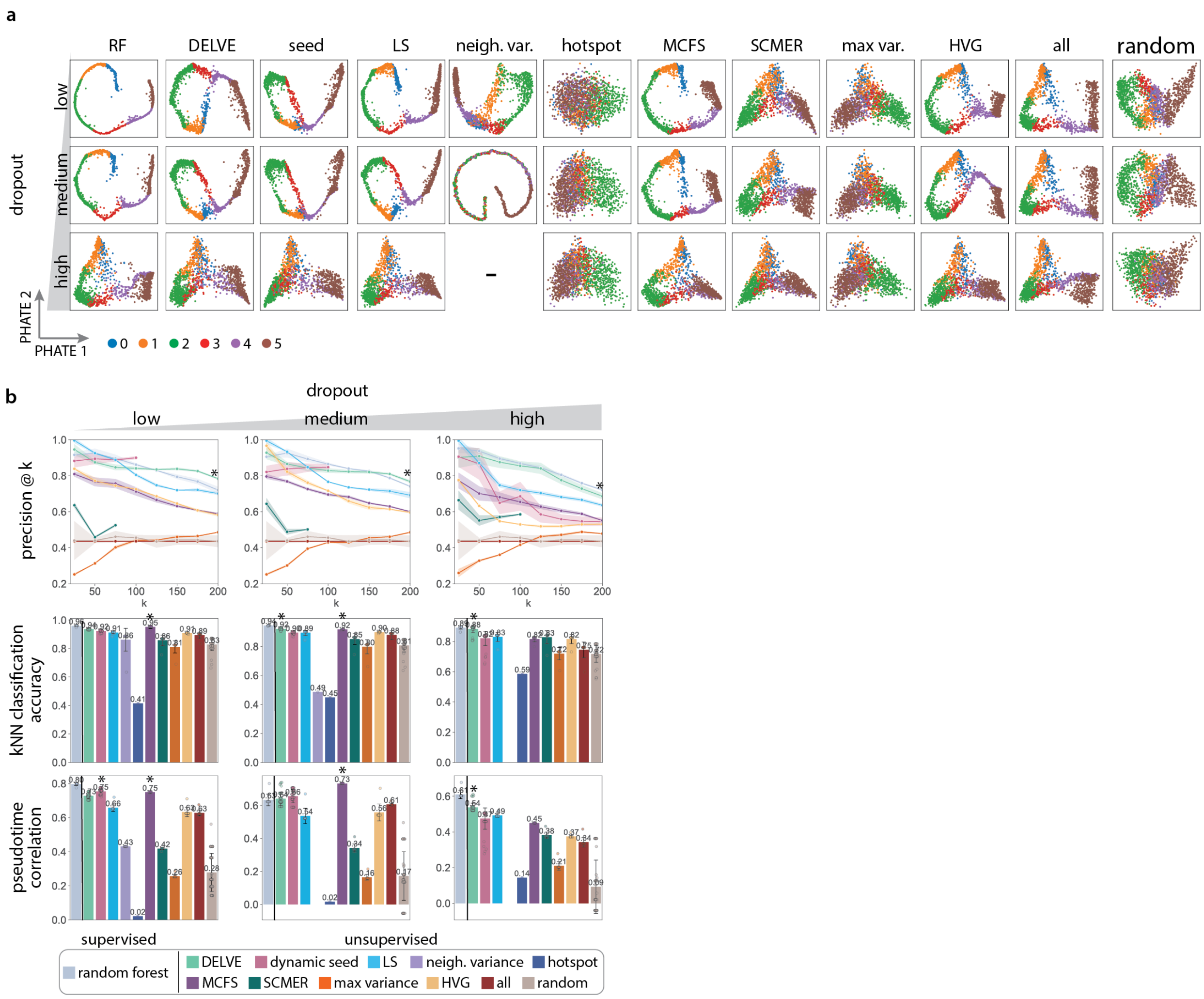

Supplementary Figure 3: Comparison of feature selection methods on preserving linear differentiation trajectories is the presence of dropout noise. Ten single-cell RNA sequencing datasets with linear trajectories (1500 cells $\times$ 500 genes) were simulated with Splatter. Feature selection performance was subsequently assessed using the top $p = 100$ ranked genes for each feature selection strategy. (a) Example PHATE visualizations of simulated linear differentiation trajectories for twelve feature selection strategies when subjected to an increase in the amount of dropout (low, medium, high). Technical dropout was simulated by undersampling mRNA counts by sampling from a binomial distribution with the scale parameter or dropout rate proportional to the mean expression of each gene (low: $\lambda = 0$, medium: $\lambda = 0.05$, low: $\lambda = 0.1$). (b) Performance of twelve different feature selection methods when subjected to an increase in the amount of dropout noise. Following feature selection ($p = 100$), trajectory preservation was quantitatively assessed according to several metrics: the precision of differentially expressed genes at k selected genes (top), k-NN classification accuracy (middle), and pseudotime correlation (bottom) across 10 random trials. Error bands represent the standard deviation over $n = 10$ simulation datasets. Barplots show the mean $\pm$ the standard deviation over $n = 10$ simulation datasets. * indicates the method with the highest median score. - indicates that the method identified no features. Source data are provided in a Source Data file.

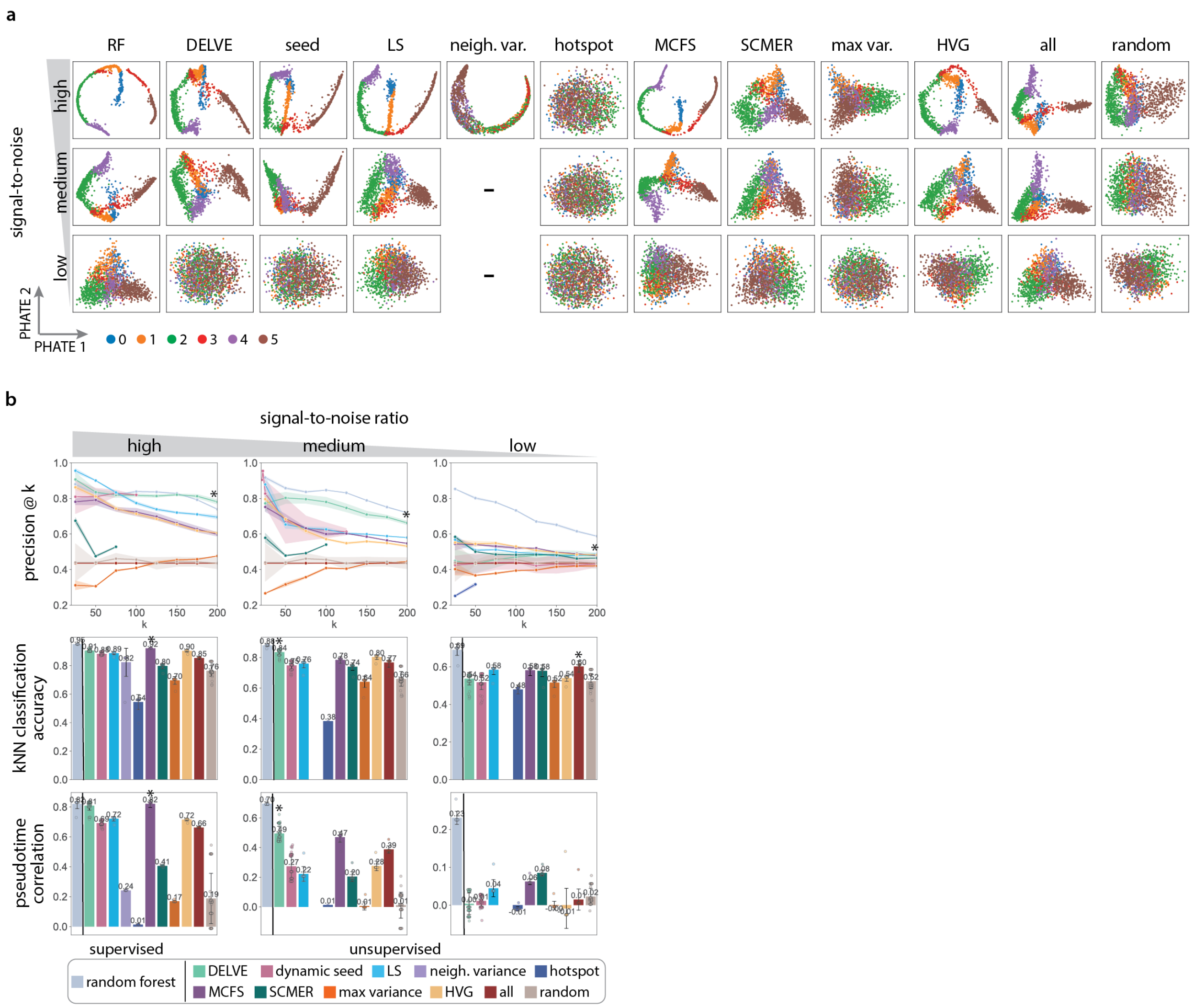

Supplementary Figure 4: Comparison of feature selection methods on preserving bifurcation differentiation trajectories under a reduction in the signal-to-noise ratio. Ten single-cell RNA sequencing datasets with bifurcation trajectories (1500 cells × 500 genes) were simulated with Splatter. Feature selection performance was subsequently assessed using the top $p = 100$ ranked genes for each feature selection strategy. (a) Example PHATE visualizations of simulated bifurcation differentiation trajectories for twelve feature selection strategies when subjected to a reduction in the signal-to-noise ratio (high, medium, low). The signal-to-noise ratio was altered by modifying the biological coefficient of variation parameter within Splatter (high: $\texttt{BCV} = 0.1$, medium: $\texttt{BCV} = 0.25$, low: $\texttt{BCV} = 0.5$). This scaling factor controls the mean-variance relationship between genes, where lowly expressed genes are more variable than highly expressed genes. (b) Performance of twelve different feature selection methods when subjected to a reduction in the signal-to-noise ratio. Following feature selection ($p = 100$), trajectory preservation was quantitatively assessed according to several metrics: the precision of differentially expressed genes at k selected genes (top), k-NN classification accuracy (middle), and pseudotime correlation (bottom) across 10 random trials. Error bands represent the standard deviation over $n = 10$ simulation datasets. Barplots show the mean ± the standard deviation over $n = 10$ simulation datasets. * indicates the method with the highest median score. - indicates that the method identified no features. Source data are provided in a Source Data file.

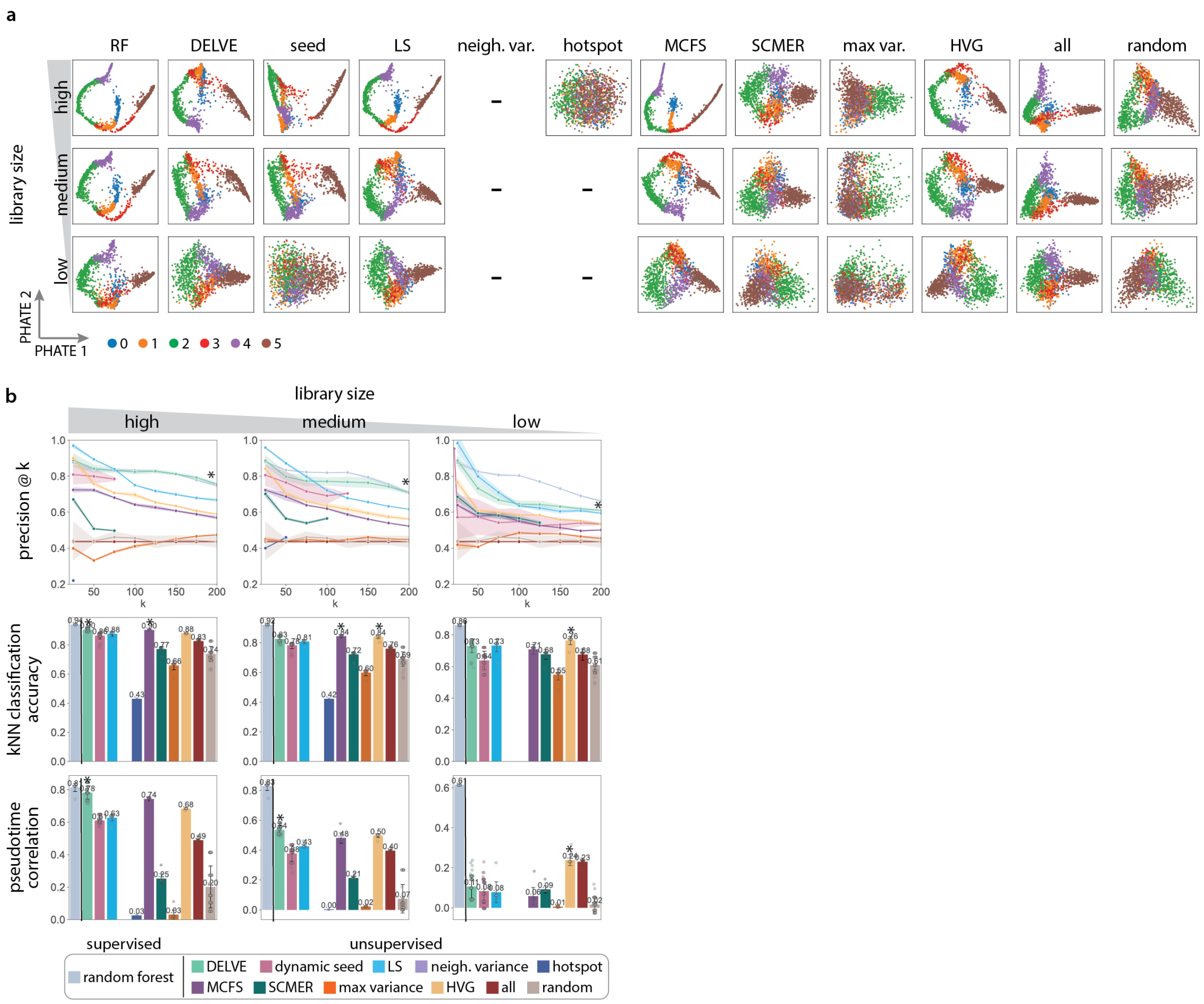

Supplementary Figure 5: Comparison of feature selection methods on preserving bifurcation differentiation trajectories in the presence of library size noise. Ten single-cell RNA sequencing datasets with bifurcation trajectories (1500 cells $\times$ 500 genes) were simulated with Splatter. Feature selection performance was subsequently assessed using the top $p = 100$ ranked genes for each feature selection strategy. (a) Example PHATE visualizations of simulated bifurcation differentiation trajectories for twelve feature selection strategies when subjected to a reduction in the total mRNA count (high, medium, low). Library size was reduced by modifying the location parameter in the log-normal distribution in Splatter that specifies library size scaling factors (high: location = 12, medium: location = 11, low: location = 10). (b) Performance of twelve different feature selection methods when subjected to a reduction in total mRNA count. Following feature selection ($p = 100$), trajectory preservation was quantitatively assessed according to several metrics: the precision of differentially expressed genes at k selected genes (top), k-NN classification accuracy (middle), and pseudotime correlation (bottom) across 10 random trials. Error bands represent the standard deviation over $n = 10$ simulation datasets. Barplots show the mean $\pm$ the standard deviation over $n = 10$ simulation datasets. * indicates the method with the highest median score. - indicates that the method identified no features. Source data are provided in a Source Data file.

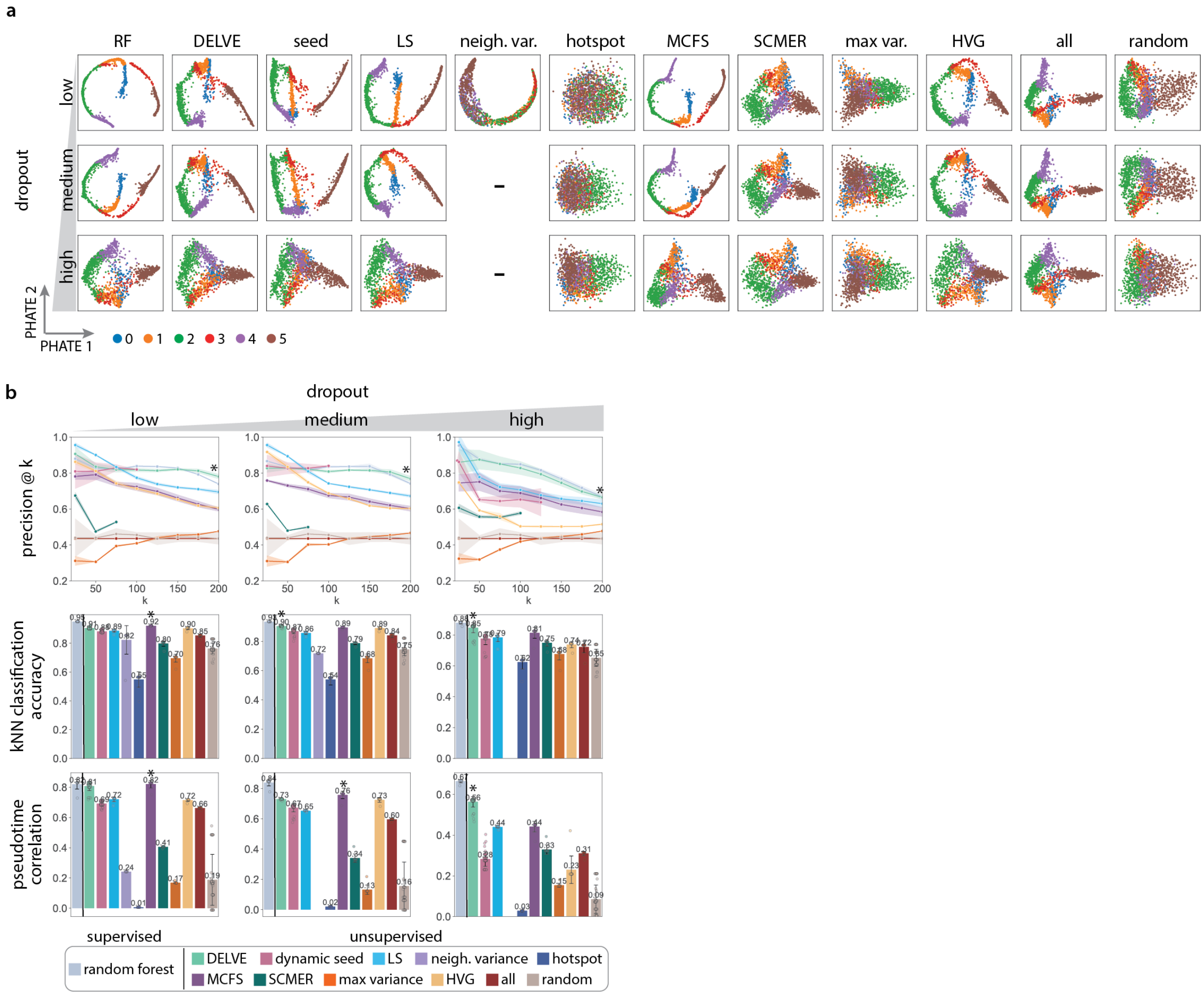

Supplementary Figure 6: Comparison of feature selection methods on preserving bifurcation differentiation trajectories is the presence of dropout noise. Ten single-cell RNA sequencing datasets with bifurcation trajectories (1500 cells $\times$ 500 genes) were simulated with Splatter. Feature selection performance was subsequently assessed using the top $p = 100$ ranked genes for each feature selection strategy. (a) Example PHATE visualizations of simulated bifurcation differentiation trajectories for twelve feature selection strategies when subjected to an increase in the amount of dropout (low, medium, high). Technical dropout was simulated by undersampling mRNA counts by sampling from a binomial distribution with the scale parameter or dropout rate proportional to the mean expression of each gene (low: $\lambda = 0$, medium: $\lambda = 0.05$, low: $\lambda = 0.1$). (b) Performance of twelve different feature selection methods when subjected to an increase in the amount of dropout noise. Following feature selection ($p = 100$), trajectory preservation was quantitatively assessed according to several metrics: the precision of differentially expressed genes at k selected genes (top), k-NN classification accuracy (middle), and pseudotime correlation (bottom) across 10 random trials. Error bands represent the standard deviation over $n = 10$ simulation datasets. Barplots show the mean $\pm$ the standard deviation over $n = 10$ simulation datasets. * indicates the method with the highest median score. - indicates that the method identified no features. Source data are provided in a Source Data file.

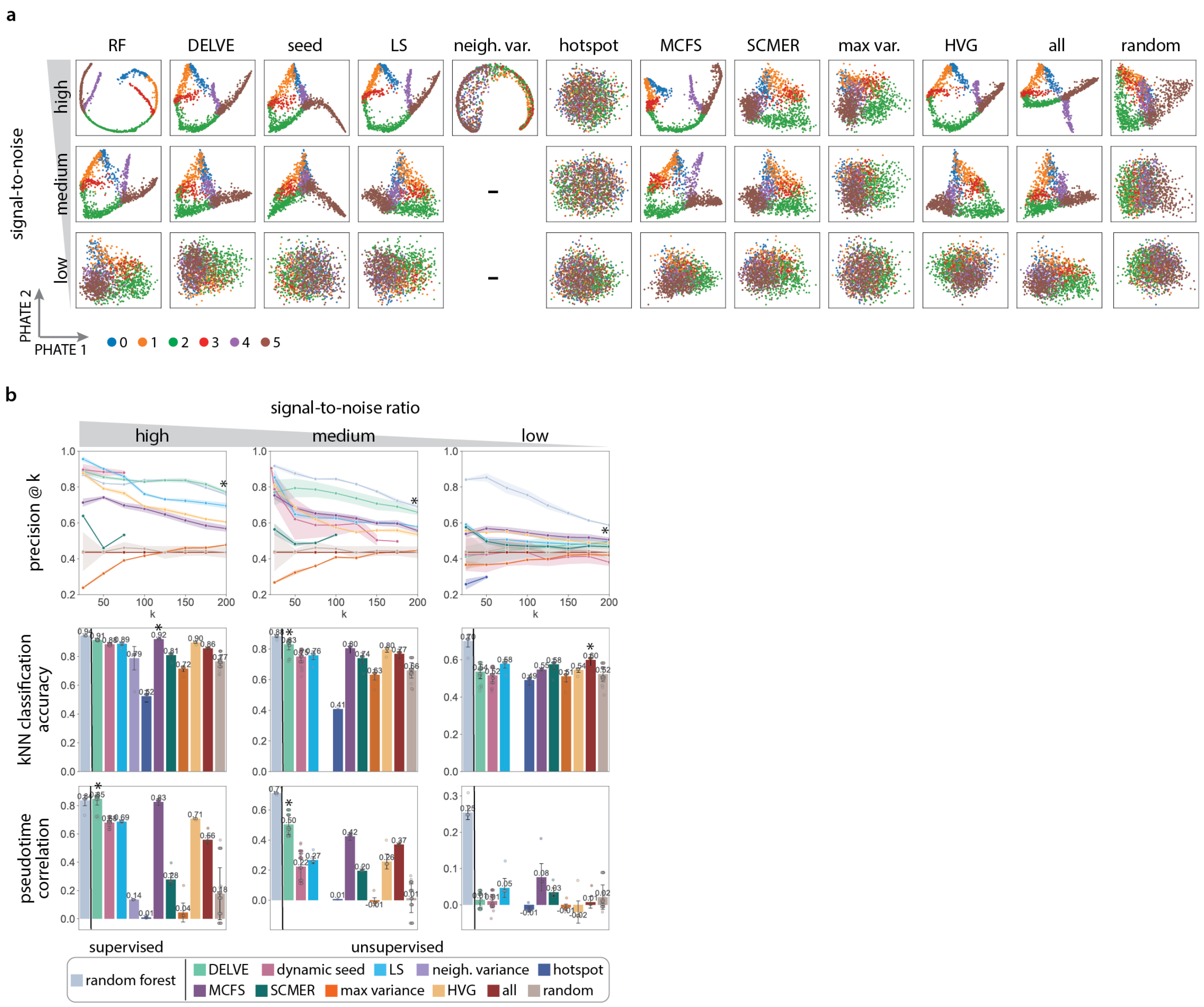

Supplementary Figure 7: Comparison of feature selection methods on preserving tree differentiation trajectories under a reduction in the signal-to-noise ratio. Ten single-cell RNA sequencing datasets with tree trajectories (1500 cells × 500 genes) were simulated with Splatter. Feature selection performance was subsequently assessed using the top $p = 100$ ranked genes for each feature selection strategy. (a) Example PHATE visualizations of simulated tree differentiation trajectories for twelve feature selection strategies when subjected to a reduction in the signal-to-noise ratio (high, medium, low). The signal-to-noise ratio was altered by modifying the biological coefficient of variation parameter within Splatter (high: $\texttt{BCV} = 0.1$, medium: $\texttt{BCV} = 0.25$, low: $\texttt{BCV} = 0.5$). This scaling factor controls the mean-variance relationship between genes, where lowly expressed genes are more variable than highly expressed genes. (b) Performance of twelve different feature selection methods when subjected to a reduction in the signal-to-noise ratio. Following feature selection ($p = 100$), trajectory preservation was quantitatively assessed according to several metrics: the precision of differentially expressed genes at k selected genes (top), k-NN classification accuracy (middle), and pseudotime correlation (bottom) across 10 random trials. Error bands represent the standard deviation over $n = 10$ simulation datasets. Barplots show the mean ± the standard deviation over $n = 10$ simulation datasets. * indicates the method with the highest median score. - indicates that the method identified no features. Source data are provided in a Source Data file.

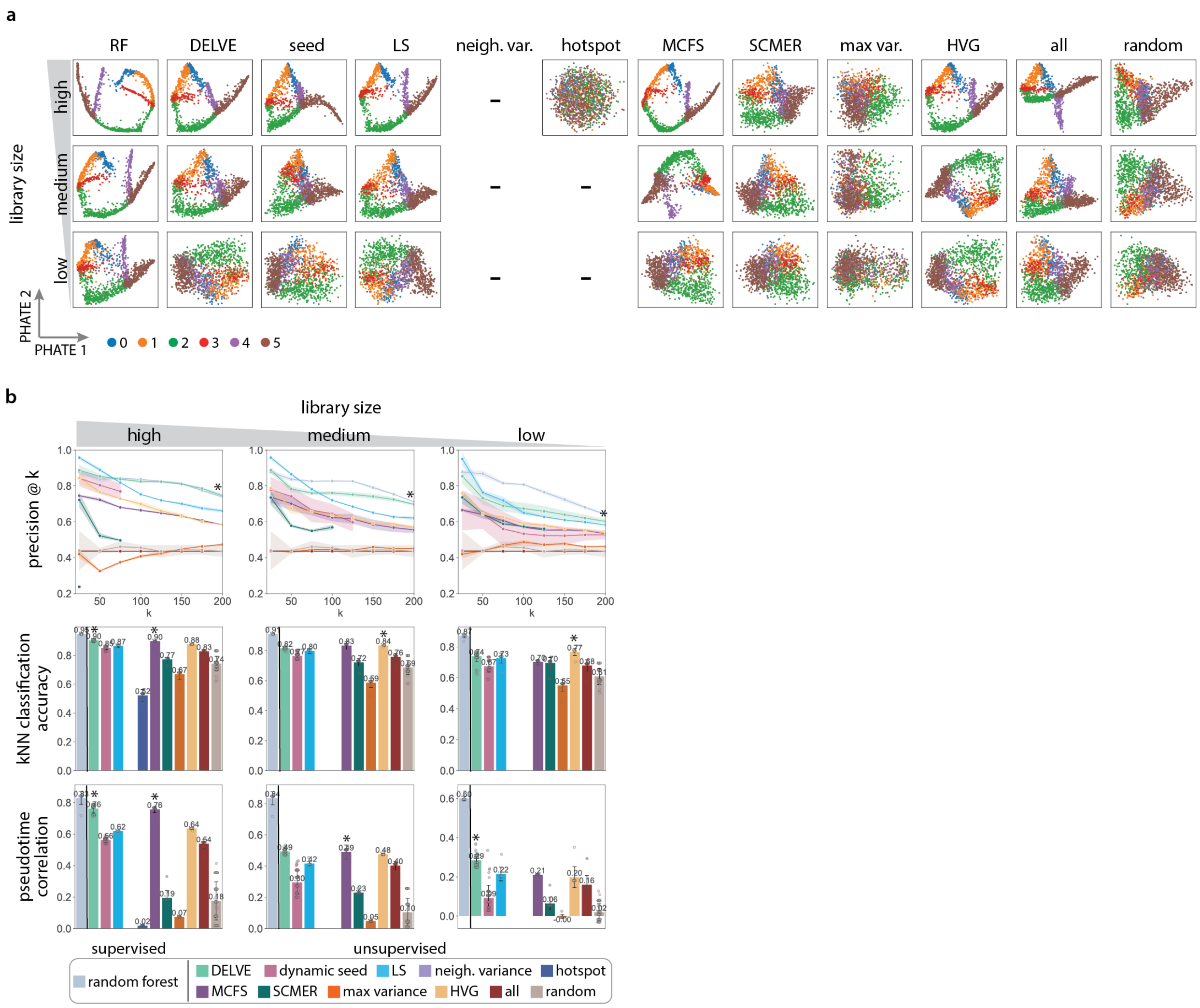

Supplementary Figure 8: Comparison of feature selection methods on preserving tree differentiation trajectories in the presence of library size noise. Ten single-cell RNA sequencing datasets with tree trajectories (1500 cells $\times$ 500 genes) were simulated with Splatter. Feature selection performance was subsequently assessed using the top $p = 100$ ranked genes for each feature selection strategy. (a) Example PHATE visualizations of simulated tree differentiation trajectories for twelve feature selection strategies when subjected to a reduction in the total mRNA count (high, medium, low). Library size was reduced by modifying the location parameter in the log-normal distribution in Splatter that specifies library size scaling factors (high: location = 12, medium: location = 11, low: location = 10). (b) Performance of twelve different feature selection methods when subjected to a reduction in total mRNA count. Following feature selection ($p = 100$), trajectory preservation was quantitatively assessed according to several metrics: the precision of differentially expressed genes at k selected genes (top), k-NN classification accuracy (middle), and pseudotime correlation (bottom) across 10 random trials. Error bands represent the standard deviation over $n = 10$ simulation datasets. Barplots show the mean $\pm$ the standard deviation over $n = 10$ simulation datasets. * indicates the method with the highest median score. - indicates that the method identified no features. Source data are provided in a Source Data file.

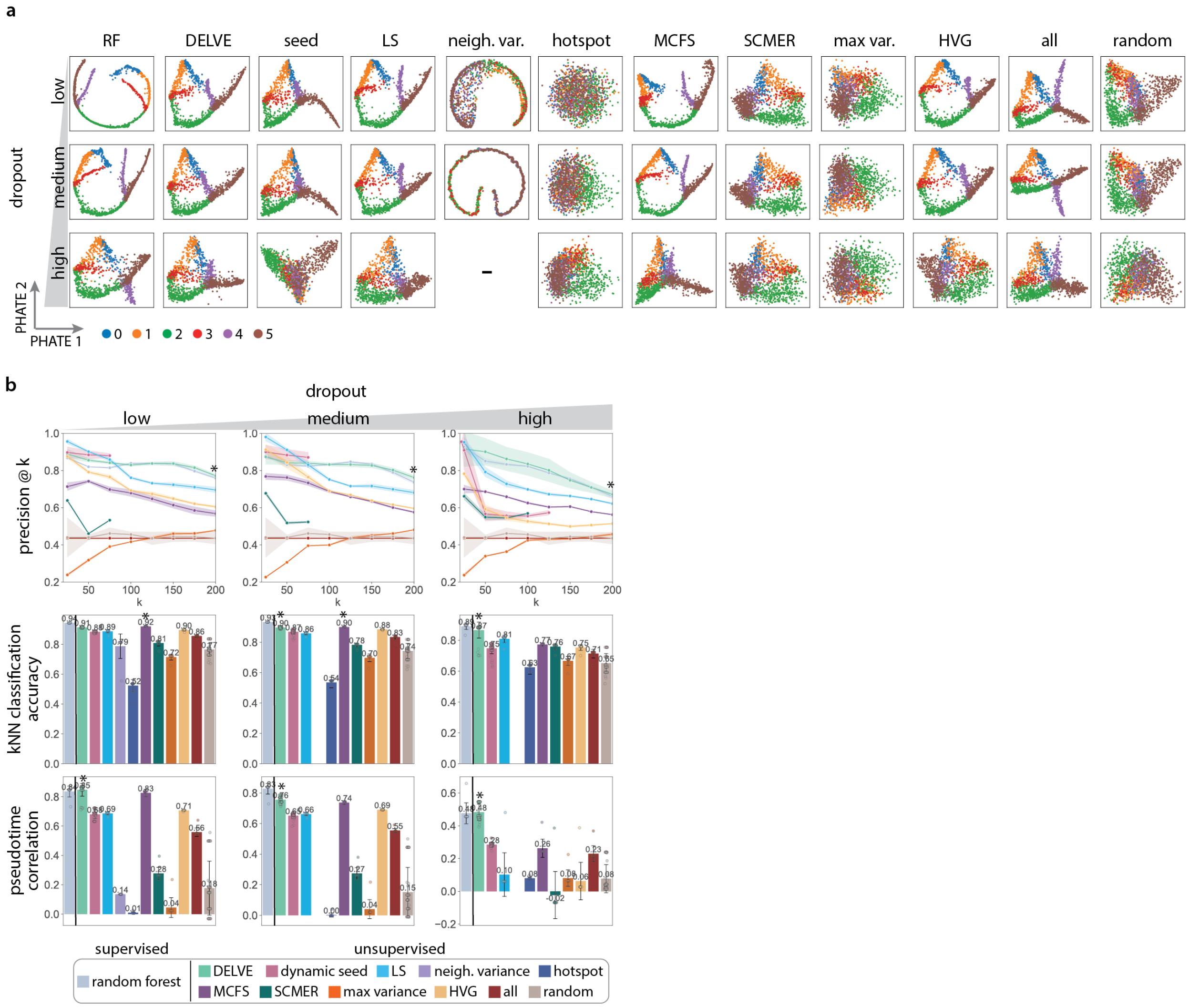

Supplementary Figure 9: Comparison of feature selection methods on preserving tree differentiation trajectories is the presence of dropout noise. Ten single-cell RNA sequencing datasets with tree trajectories (1500 cells $\times$ 500 genes) were simulated with Splatter. Feature selection performance was subsequently assessed using the top $p = 100$ ranked genes for each feature selection strategy. (a) Example PHATE visualizations of simulated tree differentiation trajectories for twelve feature selection strategies when subjected to an increase in the amount of dropout (low, medium, high). Technical dropout was simulated by undersampling mRNA counts by sampling from a binomial distribution with the scale parameter or dropout rate proportional to the mean expression of each gene (low: $\lambda = 0$, medium: $\lambda = 0.05$, low: $\lambda = 0.1$). (b) Performance of twelve different feature selection methods when subjected to an increase in the amount of dropout noise. Following feature selection ($p = 100$), trajectory preservation was quantitatively assessed according to several metrics: the precision of differentially expressed genes at k selected genes (top), k-NN classification accuracy (middle), and pseudotime correlation (bottom) across 10 random trials. Error bands represent the standard deviation over $n = 10$ simulation datasets. Barplots show the mean $\pm$ the standard deviation over $n = 10$ simulation datasets. * indicates the method with the highest median score. - indicates that the method identified no features. Source data are provided in a Source Data file.

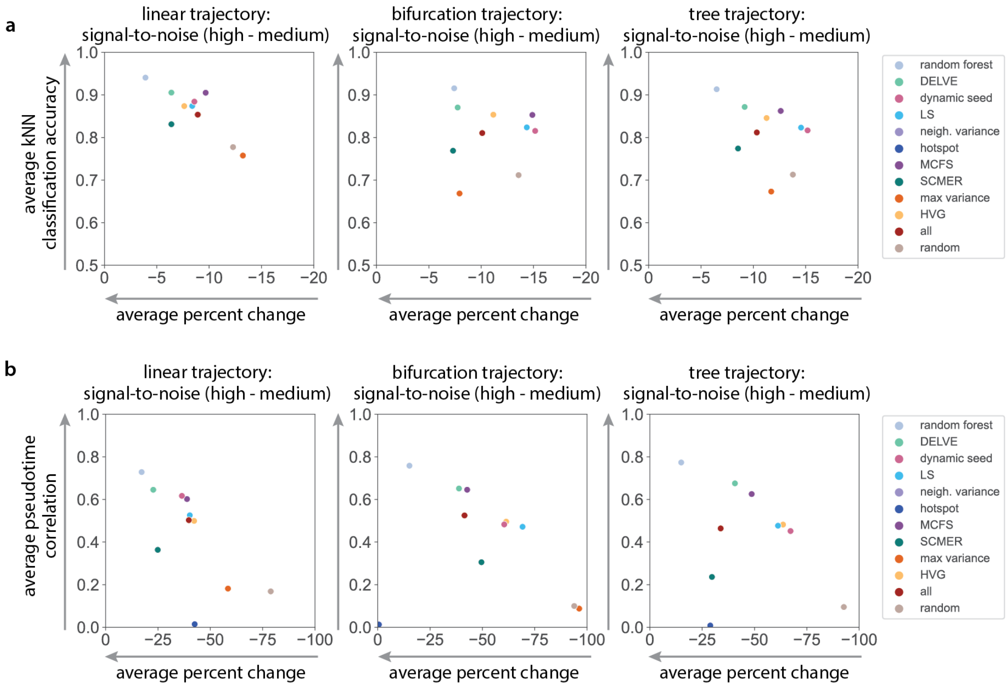

Supplementary Figure 10: Comparison of the robustness of feature selection methods on inferring differentiation trajectories under a reduction in the signal-to-noise ratio. (a) Average k-NN classification accuracy vs. average percent change in k-NN classification accuracy as the signal-to-noise ratio decreased (high to medium) and the mean-variance relationship amongst genes increased. The signal-to-noise ratio was altered by modifying the biological coefficient of variation parameter within Splatter (high: $\texttt{BCV} = 0.1$, medium: $\texttt{BCV} = 0.25$). (b) Average pseudotime correlation vs. average percent change in pseudotime correlation as the signal-to-noise ratio decreased (high to medium). Source data are provided in a Source Data file.

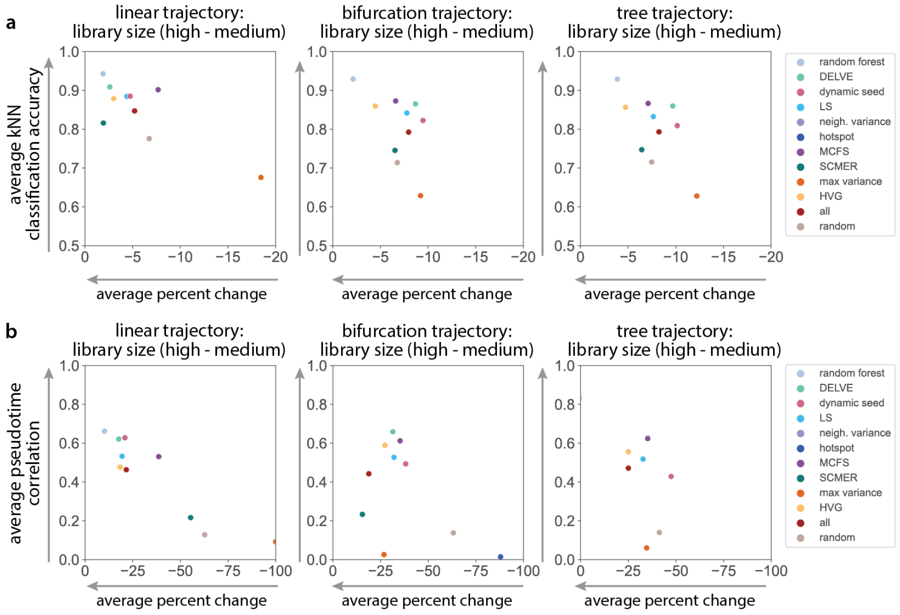

Supplementary Figure 11: Comparison of the robustness of feature selection methods on inferring differentiation trajectories under library size noise corruption. (a) Average k-NN classification accuracy vs. average percent change in k-NN classification accuracy as the total mRNA count decreased (high to medium). Library size was reduced by modifying the location parameter in the log-normal distribution in Splatter that specifies library size scaling factors (high: location = 12, medium: location = 11). (b) Average pseudotime correlation vs. average percent change in pseudotime correlation as the total mRNA count decreased (high to medium). Source data are provided in a Source Data file.

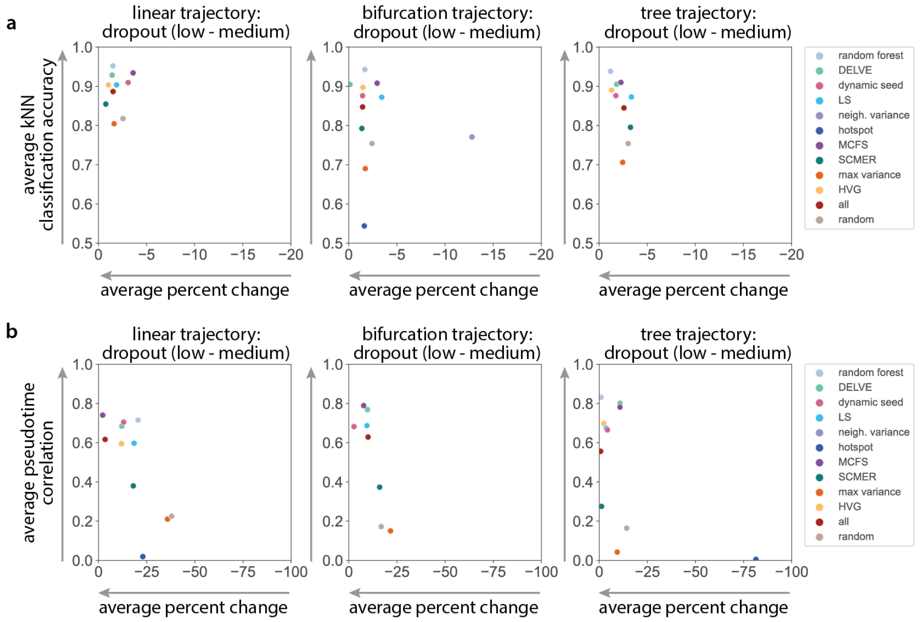

Supplementary Figure 12: Comparison of the robustness of feature selection methods on inferring differentiation trajectories under dropout noise corruption. (a) Average k-NN classification accuracy vs. average percent change in k-NN classification accuracy as the amount of dropout or sparsity increased (low to medium). Technical dropout was simulated by undersampling mRNA counts by sampling from a binomial distribution with the scale parameter or dropout rate proportional to the mean expression of each gene (low: $\lambda = 0$, medium: $\lambda = 0.05$). (b) Average pseudotime correlation vs. average percent change in pseudotime correlation as the amount of dropout or sparsity increased (low to medium). Source data are provided in a Source Data file.

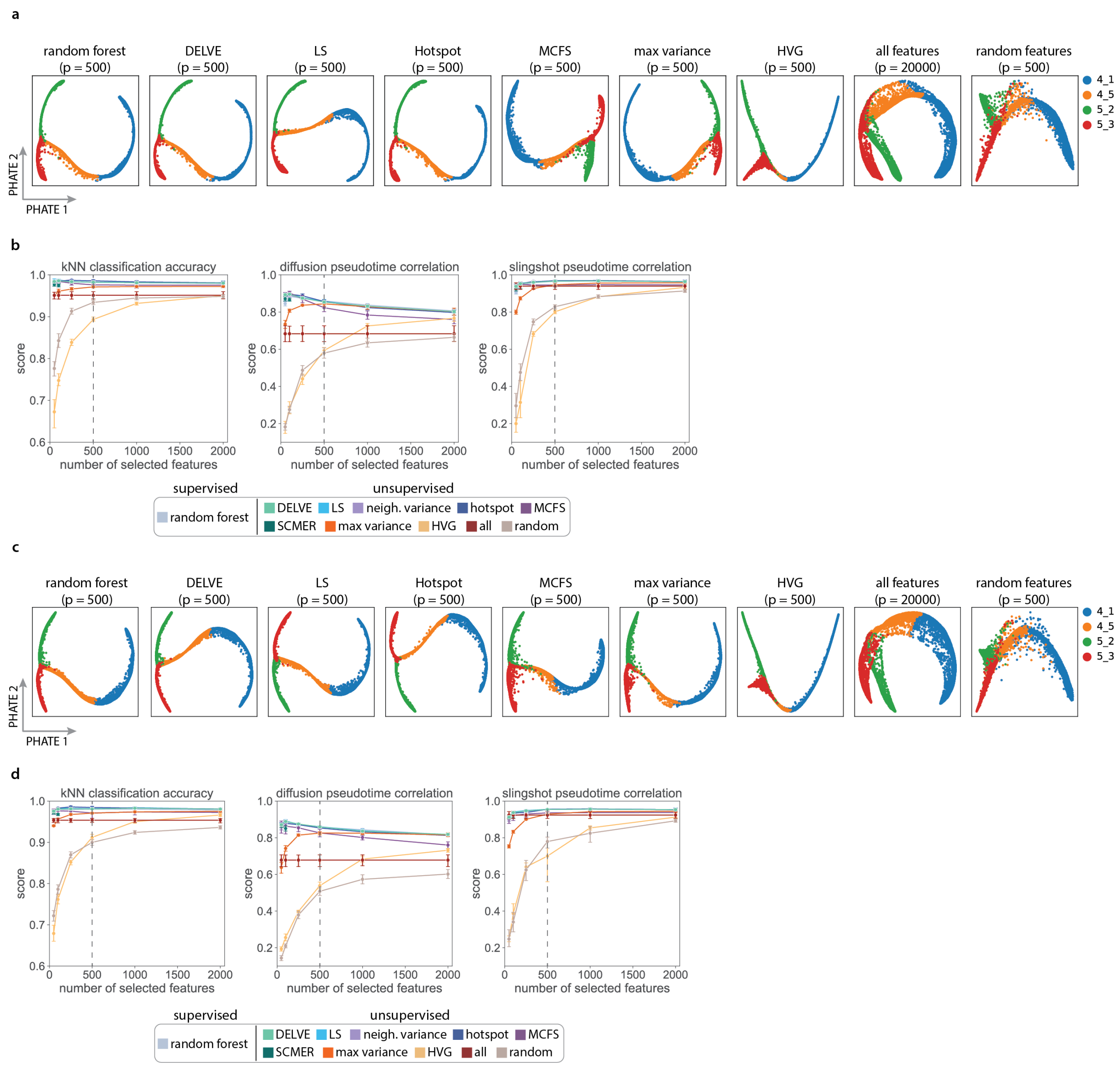

Supplementary Figure 13: Comparison of feature selection methods on preserving tree differentiation trajectories from simulated scRNA sequencing data with different mRNA capture efficiency rates. Five tree differentiation trajectories (10000 cells $\times$ 20000 genes) were simulated with SymSim by modifying the mRNA capture efficiency rate ($\alpha = 0.05, 0.04, 0.03, 0.02, 0.01$) and the within population variability (a, b) $\sigma = 0.4$ (c, d) $\sigma = 0.6$ parameters within SymSym. (a, c) Example PHATE visualizations for simulated tree differentiation trajectories for nine feature selection strategies. p indicates the number of selected features. (b, d) Performance of eleven feature selection methods on inferring tree differentiation trajectories, while modifying the number of selected features (p = 50, 100, 250, 500, 1000, 2000). Following feature selection, trajectory preservation was quantitatively assessed according to several metrics including k-NN classification accuracy, correlation between estimated cell ordering using diffusion pseudotime and the ground truth, and correlation between estimated cell ordering using Slingshot and the ground truth cell ordering. Error bars represent the standard deviation across $n = 5$ simulation datasets with different mRNA capture efficiency rates. Dashed lines highlight the performance of feature selection methods at 500 selected genes. Of note, neighborhood variance and SCMER were excluded from the PHATE visualizations as they identified fewer than 500 genes. Source data are provided in a Source Data file.

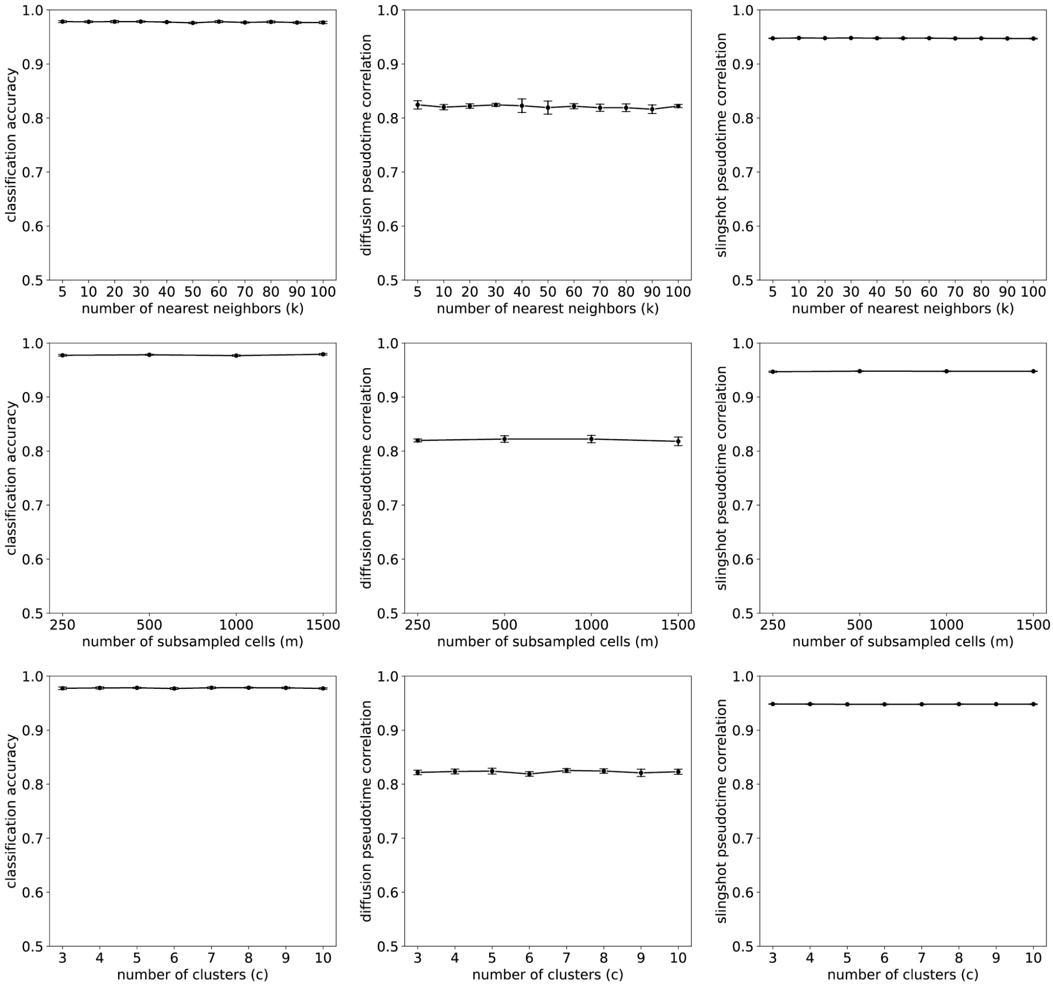

Supplementary Figure 14: DELVE is robust to changes in hyperparameters for simulated single-cell RNA sequencing data with SymSim. Single-cell RNA sequencing data (10000 cells $\times$ 20000 genes) were simulated with SymSim with a within population heterogeneity, $\sigma = 0.6$ and mRNA capture efficiency rate, $\alpha = 0.01$. DELVE achieves similar classification accuracy, diffusion pseudotime correlation, and slingshot pseudotime correlation scores across a range of hyperparameters: (top) nearest neighbors, (middle) subsampled cells, and (bottom) cluster sizes. Error bars show the standard deviation over $n = 5$ random trials, where the top 1000 genes were used for evaluation. Source data are provided in a Source Data file.

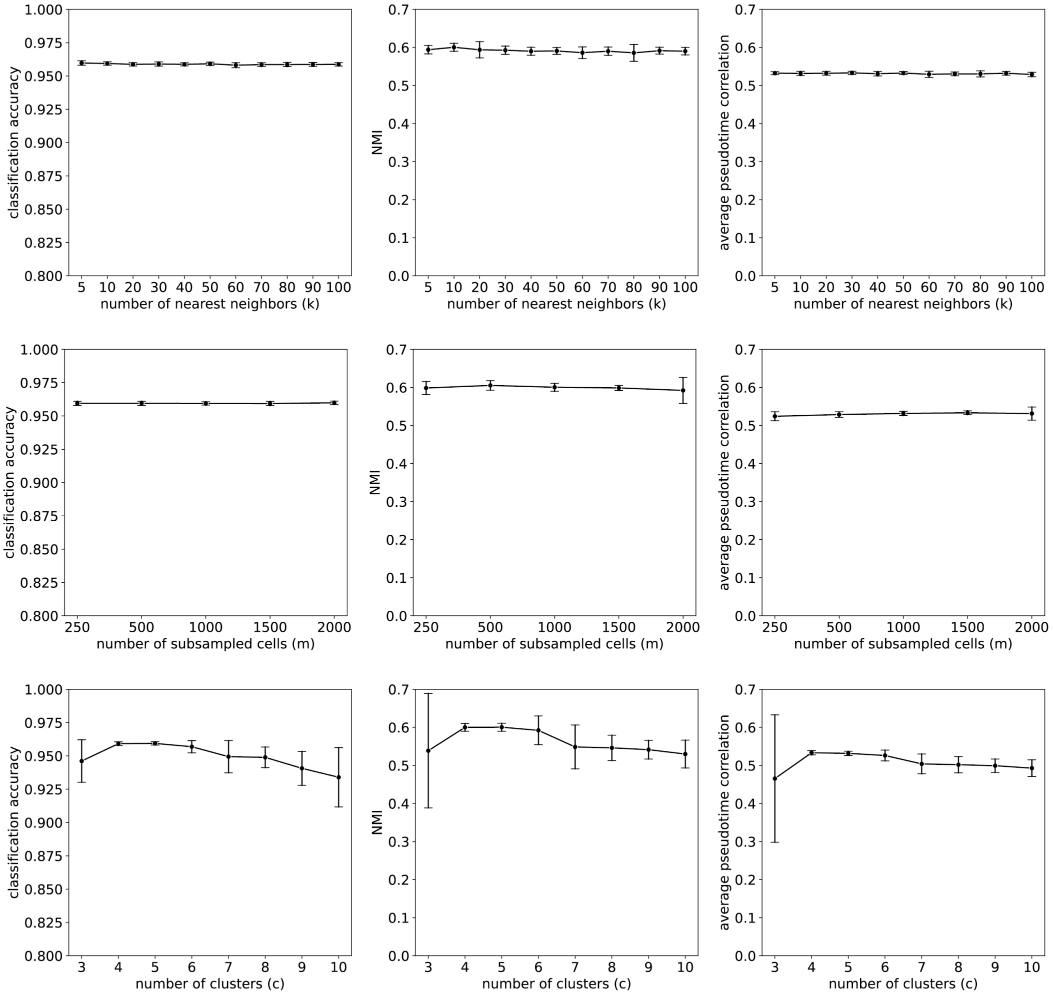

Supplementary Figure 15: DELVE is robust to changes in hyperparameters for the retinal pigmented epithelial (RPE) cell cycle dataset. DELVE achieves similar classification accuracy, normalized mutual information (NMI) clustering score, and pseudotime correlation scores across a range of hyperparameters: (top) nearest neighbors, (middle) subsampled cells, and (bottom) cluster sizes. Error bars show the standard deviation over $n = 20$ random trials, where the top 30 proteomic imaging features were used for evaluation. Source data are provided in a Source Data file.

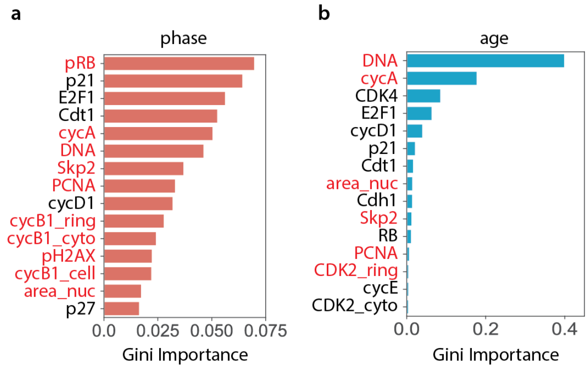

Supplementary Figure 16: Validation of DELVE seed selection on retinal pigmented epithelial (RPE) cell cycle dataset. Top ranked features identified by a (a) random forest classifier trained on ground truth cell cycle phase annotations or (b) random forest regressor trained on ground truth cell cycle age measurements. Features highlighted in red were also identified by DELVE seed selection (See Figure 4a heatmap). Source data are provided in a Source Data file.

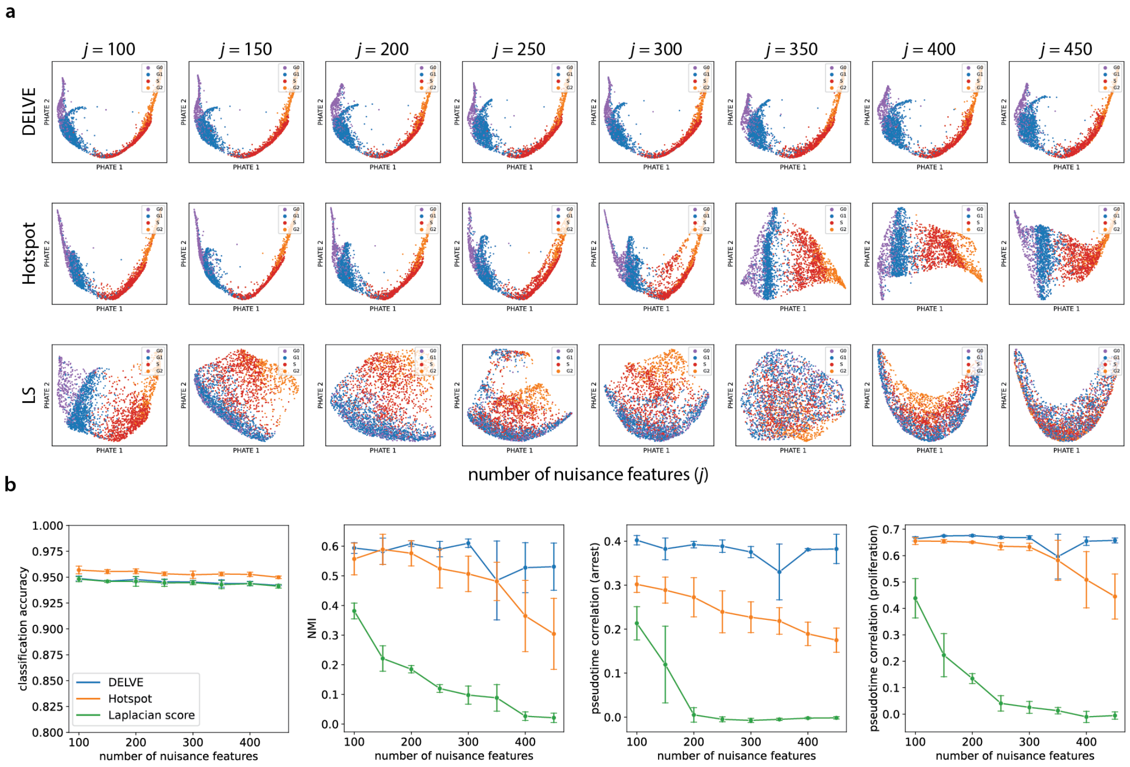

Supplementary Figure 17: Comparison of similarity-based feature selection methods on inferring RPE cell cycle trajectories with increasing amounts of noisy imaging features. To evaluate the performance of similarity-based feature selection methods on preserving cellular trajectories under increasing amounts of noisy imaging-derived features, nonsense features were added to the original dataset by randomly sampling features from the 4i RPE cycle dataset with replacement. (a) PHATE visualizations of RPE cell cycle trajectories for three similarity-based feature selection methods (DELVE, Hotspot, Laplacian Score (LS)) when subjected to increasing amounts of nonsense imaging-derived features (j = 100 to 450). (b) Performance of similarity-based feature selection methods on preserving the underlying RPE cell cycle trajectory when subjected to increasing amounts of noisy imaging-derived features. Following feature selection, trajectory preservation was quantitatively assessed according to several metrics, including classification accuracy, normalized mutual information (NMI) clustering score, and the Kendall rank correlation between estimated pseudotime and the ground truth age measurements from time-lapse imaging. Error bars show the standard deviation across $n = 5$ random trials. Source data are provided in a Source Data file.

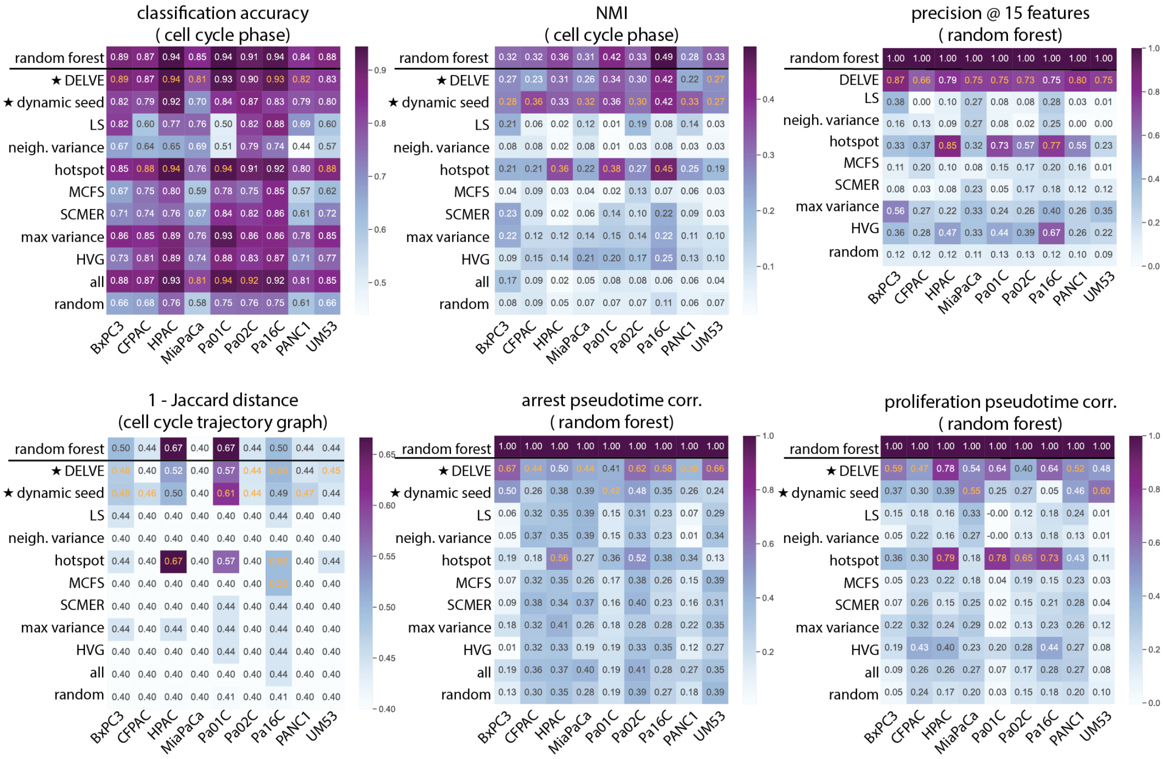

Supplementary Figure 18: Feature selection method performance on pancreatic adenocarcinoma (PDAC) cell cycle datasets. Performance of twelve feature selection methods on preserving cell cycle trajectories from 9 PDAC cancer cell lines (BxPC3, CFPAC, HPAC, MiaPaCa, Pa02C, Pa01C, Pa16C, PANC1, and UM53) profiled with protein immunofluorescence imaging. Following feature selection, cell cycle preservation was quantitatively assessed according to several metrics including: support vector machine classification accuracy to the ground truth phase annotations, normalized mutual information (NMI) clustering score to ground truth phase annotations, precision of cell cycle phase-specific imaging-derived features as measured by a random forest classifier trained on ground truth phase annotations, Jaccard distance between predicted cell cycle trajectory graphs and a ground truth reference cell cycle trajectory curated from the literature, and the Kendall rank correlation between estimated pseudotime and the ground truth as measured by a random forest classifier trained on ground truth phase annotations. Heatmaps show the average performance. Approaches with the highest average score are highlighted in yellow. Source data are provided in a Source Data file.

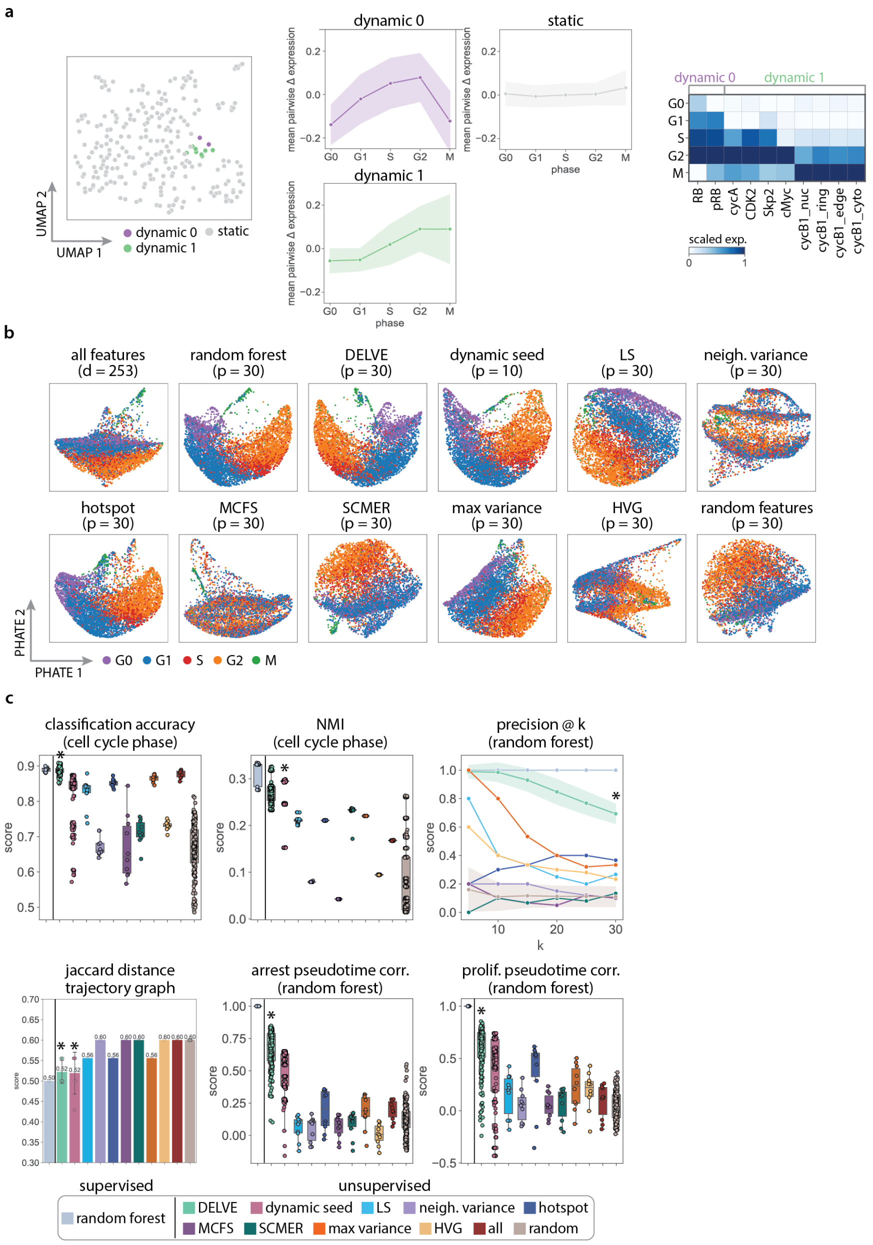

Supplementary Figure 19: DELVE recovers BxPC3 pancreatic adenocarcinoma cell cycle trajectories in protein immunofluorescence imaging data. BxPC3 cells were profiled with protein immunofluorescence imaging to measure 63 core cell cycle effectors resulting in a dataset with $d = 253$ imaging-derived features. (a) DELVE identified two modules of dynamic features representing a minimum cell cycle. (a left) UMAP visualization of image-derived features where each point indicates a dynamic or static feature identified by the model. (a middle) The average pairwise change in expression for features within a module ordered across ground truth cell cycle phase annotations. Error bands represent the standard deviation. (a right) Heatmap illustrating the standardized average expression of dynamic seed features across cell cycle phases. (b) Feature selection was performed to select the top $p = 30$ ranked features. Example PHATE visualizations of cell cycle trajectories for twelve feature selection approaches. (c) Quantitative assessment of twelve feature selection methods on preserving cell cycle phases and phase transitions according to several metrics including: support vector machine classification accuracy to the ground truth phase annotations, normalized mutual information (NMI) clustering score to ground truth phase annotations, precision of cell cycle phase-specific imaging-derived features as measured by a random forest classifier trained on ground truth phase annotations, Jaccard distance between predicted cell cycle trajectory graphs and a ground truth reference cell cycle trajectory curated from the literature, and the Kendall rank correlation between estimated pseudotime and the ground truth as measured by a random forest classifier trained on ground truth phase annotations. All error bands represent the standard deviation. All boxplots show the median (middle line), the interquartile range (upper and lower bounds of the box), and the minimum and maximum of the distributions (whiskers) over $n = 10$ random splits, seeds, or root cells. DELVE, dynamic seed, and random feature selection were run over $n = 20$ random trials to show reproducibility of the approach. * indicates the method with the highest median score. Source data are provided in a Source Data file.

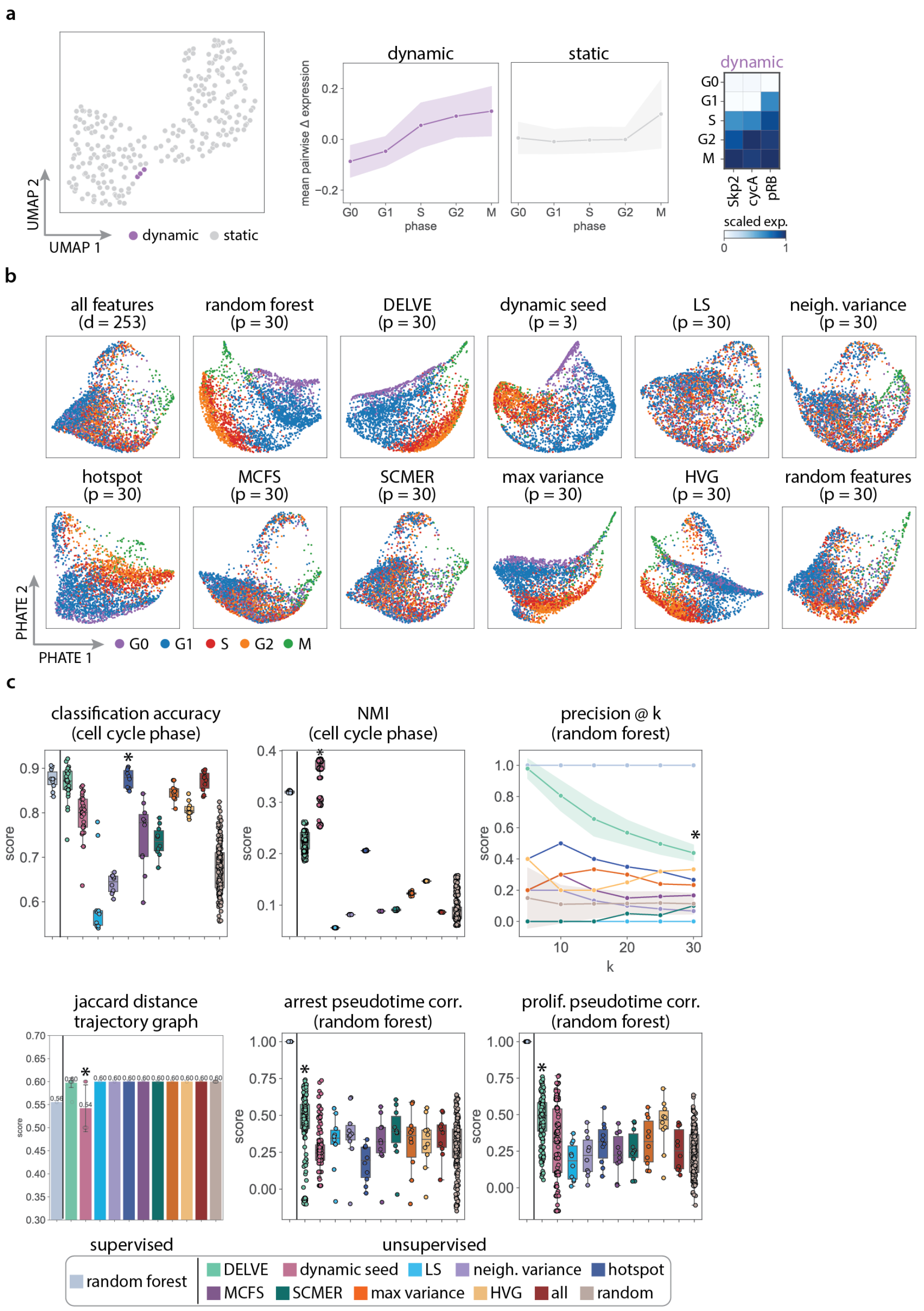

Supplementary Figure 20: DELVE recovers CFPAC pancreatic adenocarcinoma cell cycle trajectories in protein immunofluorescence imaging data. CFPAC cells were profiled with protein immunofluorescence imaging to measure 63 core cell cycle effectors resulting in a dataset with $d = 253$ imaging-derived features. (a) DELVE identified one module of dynamic features representing a minimum cell cycle. (a left) UMAP visualization of image-derived features where each point indicates a dynamic or static feature identified by the model. (a middle) The average pairwise change in expression for features within a module ordered across ground truth cell cycle phase annotations. (a right) Heatmap illustrating the standardized average expression of dynamic seed features across cell cycle phases. (b) Feature selection was performed to select the top $p = 30$ ranked features. Example PHATE visualizations of cell cycle trajectories for twelve feature selection approaches. (c) Quantitative assessment of twelve feature selection methods on preserving cell cycle phases and phase transitions according to several metrics including: support vector machine classification accuracy to the ground truth phase annotations, normalized mutual information (NMI) clustering score to ground truth phase annotations, precision of cell cycle phase-specific imaging-derived features as measured by a random forest classifier trained on ground truth phase annotations, Jaccard distance between predicted cell cycle trajectory graphs and a ground truth reference cell cycle trajectory curated from the literature, and the Kendall rank correlation between estimated pseudotime and the ground truth as measured by a random forest classifier trained on ground truth phase annotations. All error bands represent the standard deviation. All boxplots show the median (middle line), the interquartile range (upper and lower bounds of the box), and the minimum and maximum of the distributions (whiskers) over $n = 10$ random splits, seeds, or root cells. DELVE, dynamic seed, and random feature selection were run over $n = 20$ random trials to show reproducibility of the approach. * indicates the method with the highest median score. Source data are provided in a Source Data file.

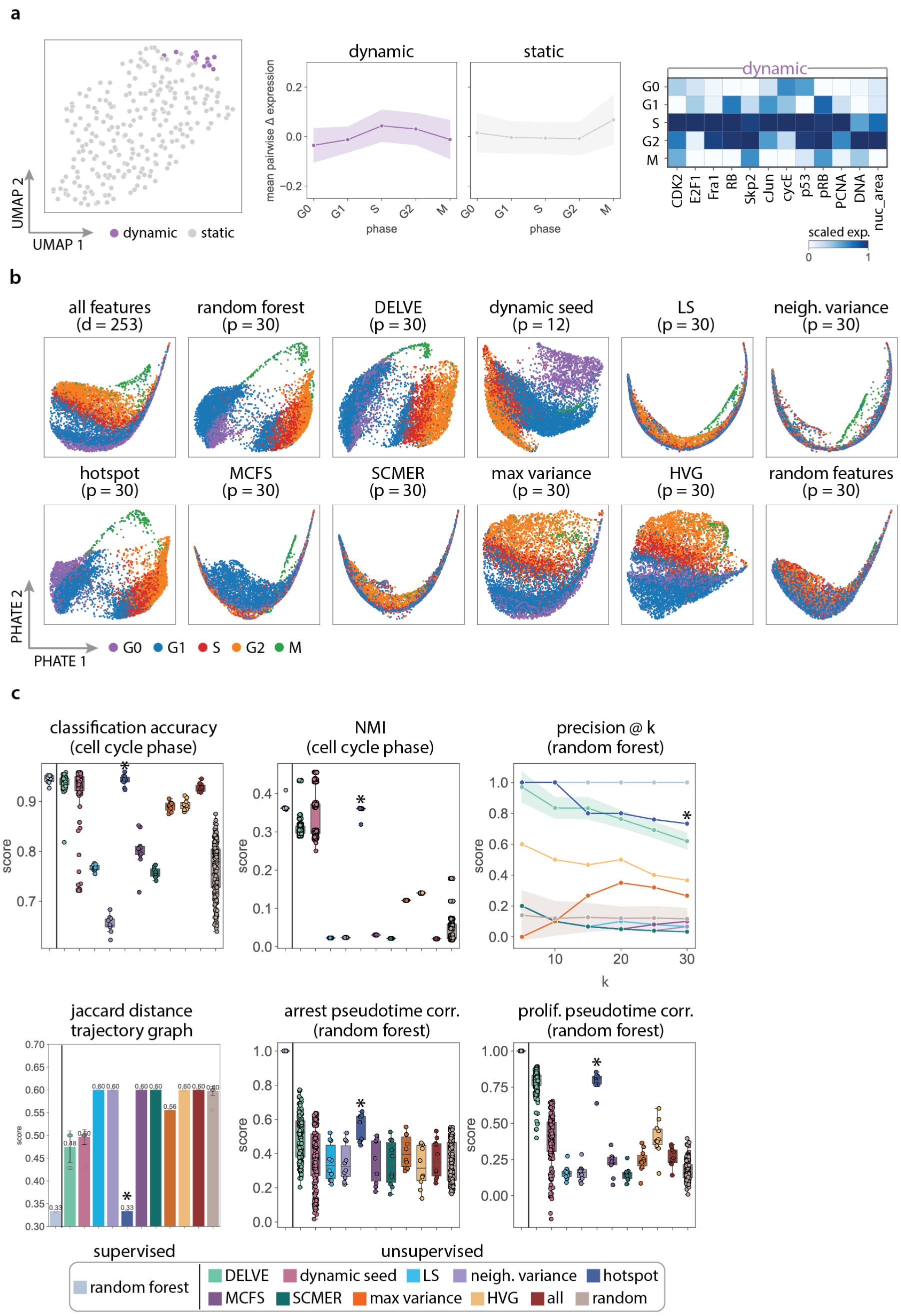

Supplementary Figure 21: DELVE recovers HPAC pancreatic adenocarcinoma cell cycle trajectories in protein immunofluorescence imaging data. HPAC cells were profiled with protein immunofluorescence imaging to measure 63 core cell cycle effectors resulting in a dataset with $d = 253$ imaging-derived features. (a) DELVE identified one module of dynamic features representing a minimum cell cycle. (a left) UMAP visualization of image-derived features where each point indicates a dynamic or static feature identified by the model. (a middle) The average pairwise change in expression for features within a module ordered across ground truth cell cycle phase annotations. (a right) Heatmap illustrating the standardized average expression of dynamic seed features across cell cycle phases. (b) Feature selection was performed to select the top $p = 30$ ranked features. Example PHATE visualizations of cell cycle trajectories for twelve feature selection approaches. (c) Quantitative assessment of twelve feature selection methods on preserving cell cycle phases and phase transitions according to several metrics including: support vector machine classification accuracy to the ground truth phase annotations, normalized mutual information (NMI) clustering score to ground truth phase annotations, precision of cell cycle phase-specific imaging-derived features as measured by a random forest classifier trained on ground truth phase annotations, Jaccard distance between predicted cell cycle trajectory graphs and a ground truth reference cell cycle trajectory curated from the literature, and the Kendall rank correlation between estimated pseudotime and the ground truth as measured by a random forest classifier trained on ground truth phase annotations. All error bands represent the standard deviation. All boxplots show the median (middle line), the interquartile range (upper and lower bounds of the box), and the minimum and maximum of the distributions (whiskers) over $n = 10$ random splits, seeds, or root cells. DELVE, dynamic seed, and random feature selection were run over $n = 20$ random trials to show reproducibility of the approach. * indicates the method with the highest median score. Source data are provided in a Source Data file.

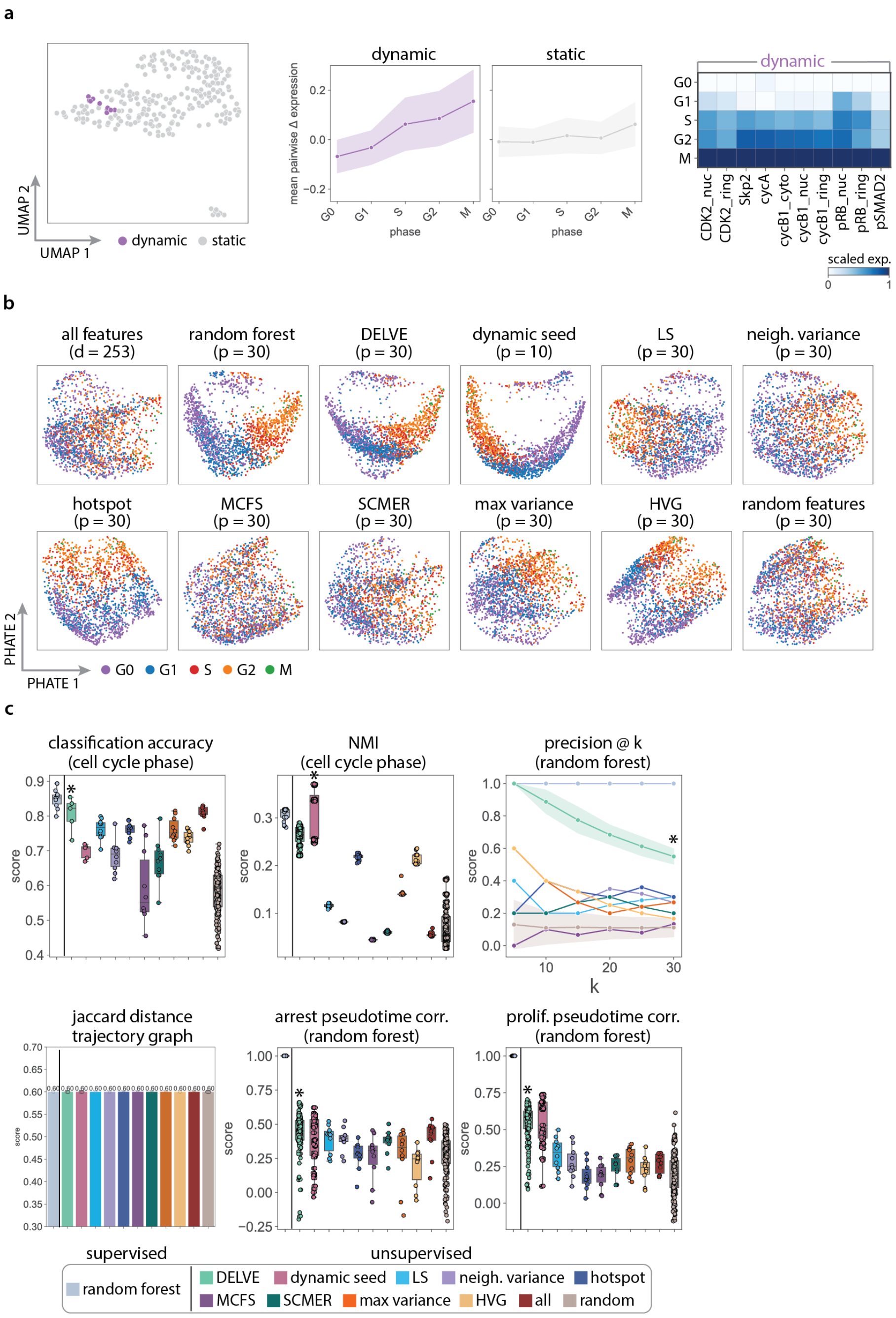

Supplementary Figure 22: DELVE recovers MiaPaCa pancreatic adenocarcinoma cell cycle trajectories in protein immunofluorescence imaging data. MiaPaCa cells were profiled with protein immunofluorescence imaging to measure 63 core cell cycle effectors resulting in a dataset with $d = 253$ imaging-derived features. (a) DELVE identified one module of dynamic features representing a minimum cell cycle. (a left) UMAP visualization of image-derived features where each point indicates a dynamic or static feature identified by the model. (a middle) The average pairwise change in expression for features within a module ordered across ground truth cell cycle phase annotations. (a right) Heatmap illustrating the standardized average expression of dynamic seed features across cell cycle phases. (b) Feature selection was performed to select the top $p = 30$ ranked features. Example PHATE visualizations of cell cycle trajectories for twelve feature selection approaches. (c) Quantitative assessment of twelve feature selection methods on preserving cell cycle phases and phase transitions according to several metrics including: support vector machine classification accuracy to the ground truth phase annotations, normalized mutual information (NMI) clustering score to ground truth phase annotations, precision of cell cycle phase-specific imaging-derived features as measured by a random forest classifier trained on ground truth phase annotations, Jaccard distance between predicted cell cycle trajectory graphs and a ground truth reference cell cycle trajectory curated from the literature, and the Kendall rank correlation between estimated pseudotime and the ground truth as measured by a random forest classifier trained on ground truth phase annotations. All error bands represent the standard deviation. All boxplots show the median (middle line), the interquartile range (upper and lower bounds of the box), and the minimum and maximum of the distributions (whiskers) over $n = 10$ random splits, seeds, or root cells. DELVE, dynamic seed, and random feature selection were run over $n = 20$ random trials to show reproducibility of the approach. * indicates the method with the highest median score. Source data are provided in a Source Data file.

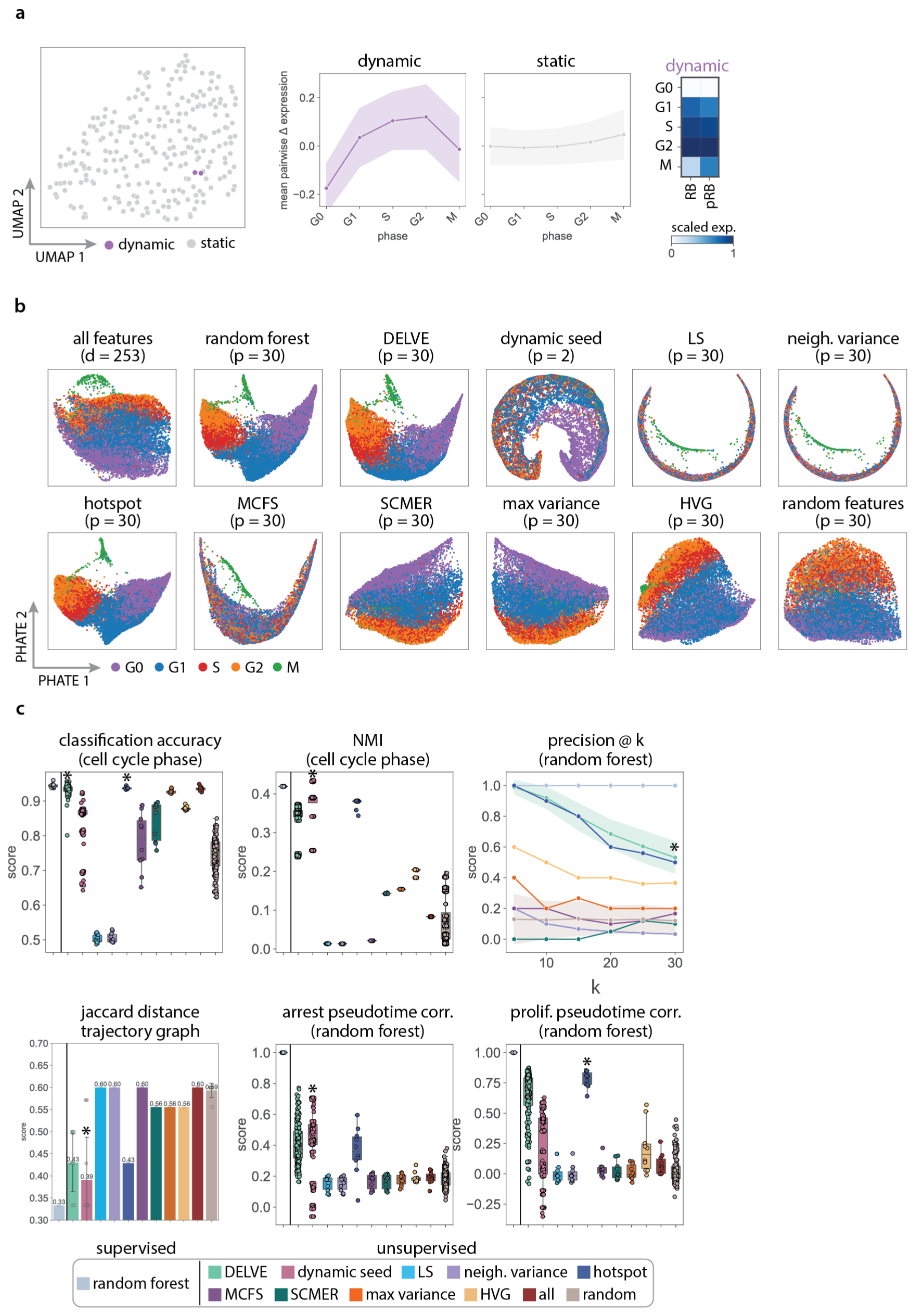

Supplementary Figure 23: DELVE recovers Pa01C pancreatic adenocarcinoma cell cycle trajectories in protein immunofluorescence imaging data. Pa01C cells were profiled with protein immunofluorescence imaging to measure 63 core cell cycle effectors resulting in a dataset with $d = 253$ imaging-derived features. (a) DELVE identified one module of dynamic features representing a minimum cell cycle. (a left) UMAP visualization of image-derived features where each point indicates a dynamic or static feature identified by the model. (a middle) The average pairwise change in expression for features within a module ordered across ground truth cell cycle phase annotations. (a right) Heatmap illustrating the standardized average expression of dynamic seed features across cell cycle phases. (b) Feature selection was performed to select the top $p = 30$ ranked features. Example PHATE visualizations of cell cycle trajectories for twelve feature selection approaches. (c) Quantitative assessment of twelve feature selection methods on preserving cell cycle phases and phase transitions according to several metrics including: support vector machine classification accuracy to the ground truth phase annotations, normalized mutual information (NMI) clustering score to ground truth phase annotations, precision of cell cycle phase-specific imaging-derived features as measured by a random forest classifier trained on ground truth phase annotations, Jaccard distance between predicted cell cycle trajectory graphs and a ground truth reference cell cycle trajectory curated from the literature, and the Kendall rank correlation between estimated pseudotime and the ground truth as measured by a random forest classifier trained on ground truth phase annotations.All error bands represent the standard deviation. All boxplots show the median (middle line), the interquartile range (upper and lower bounds of the box), and the minimum and maximum of the distributions (whiskers) over $n = 10$ random splits, seeds, or root cells. DELVE, dynamic seed, and random feature selection were run over $n = 20$ random trials to show reproducibility of the approach. * indicates the method with the highest median score. Source Data are provided in a Source Data file.

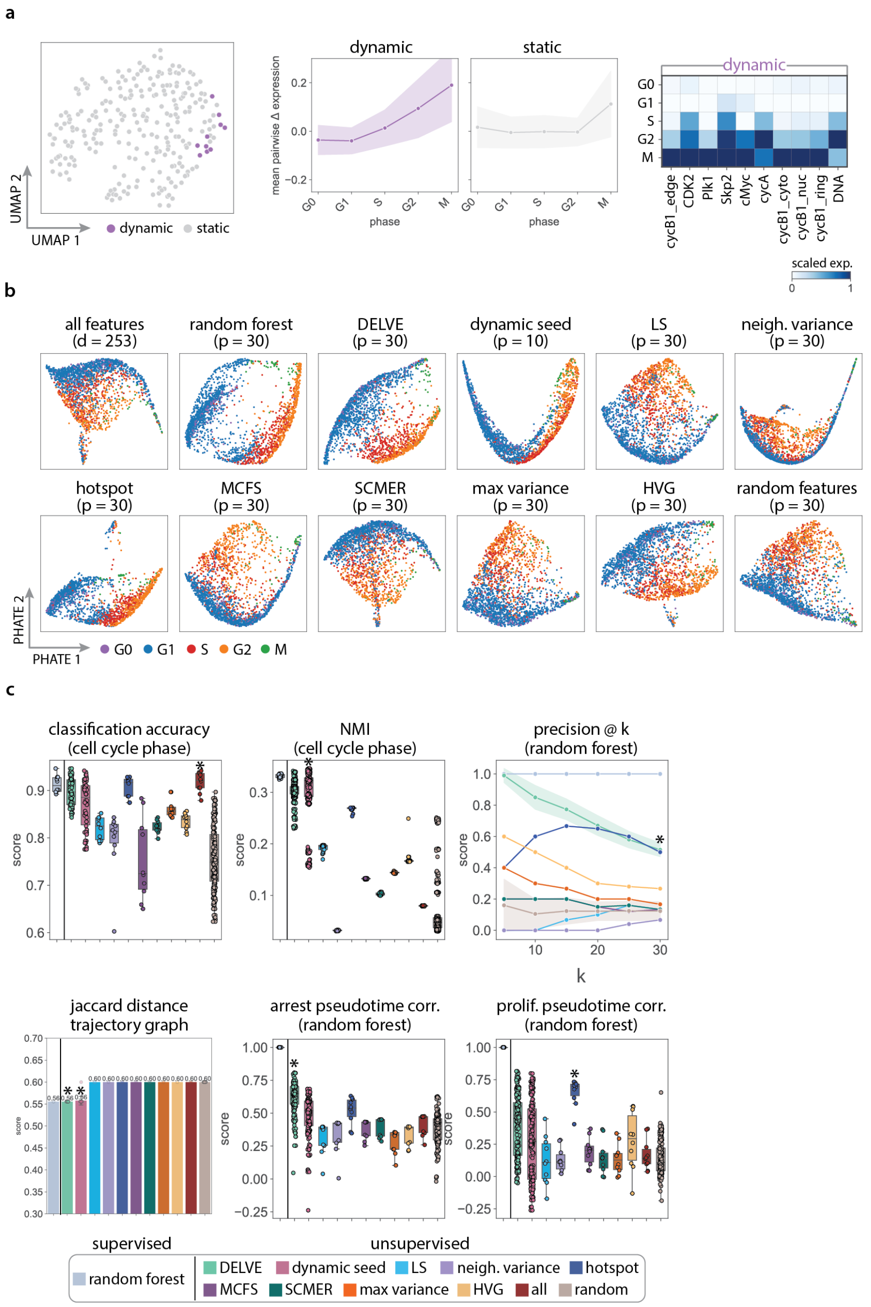

Supplementary Figure 24: DELVE recovers Pa02C pancreatic adenocarcinoma cell cycle trajectories in protein immunofluorescence imaging data. Pa02C cells were profiled with protein immunofluorescence imaging to measure 63 core cell cycle effectors resulting in a dataset with $d = 253$ imaging-derived features. (a) DELVE identified one module of dynamic features representing a minimum cell cycle. (a left) UMAP visualization of image-derived features where each point indicates a dynamic or static feature identified by the model. (a middle) The average pairwise change in expression for features within a module ordered across ground truth cell cycle phase annotations. (a right) Heatmap illustrating the standardized average expression of dynamic seed features across cell cycle phases. (b) Feature selection was performed to select the top $p = 30$ ranked features. Example PHATE visualizations of cell cycle trajectories for twelve feature selection approaches. (c) Quantitative assessment of twelve feature selection methods on preserving cell cycle phases and phase transitions according to several metrics including: support vector machine classification accuracy to the ground truth phase annotations, normalized mutual information (NMI) clustering score to ground truth phase annotations, precision of cell cycle phase-specific imaging-derived features as measured by a random forest classifier trained on ground truth phase annotations, Jaccard distance between predicted cell cycle trajectory graphs and a ground truth reference cell cycle trajectory curated from the literature, and the Kendall rank correlation between estimated pseudotime and the ground truth as measured by a random forest classifier trained on ground truth phase annotations. All error bands represent the standard deviation. All boxplots show the median (middle line), the interquartile range (upper and lower bounds of the box), and the minimum and maximum of the distributions (whiskers) over $n = 10$ random splits, seeds, or root cells. DELVE, dynamic seed, and random feature selection were run over $n = 20$ random trials to show reproducibility of the approach. * indicates the method with the highest median score. Source data are provided in a Source Data file.

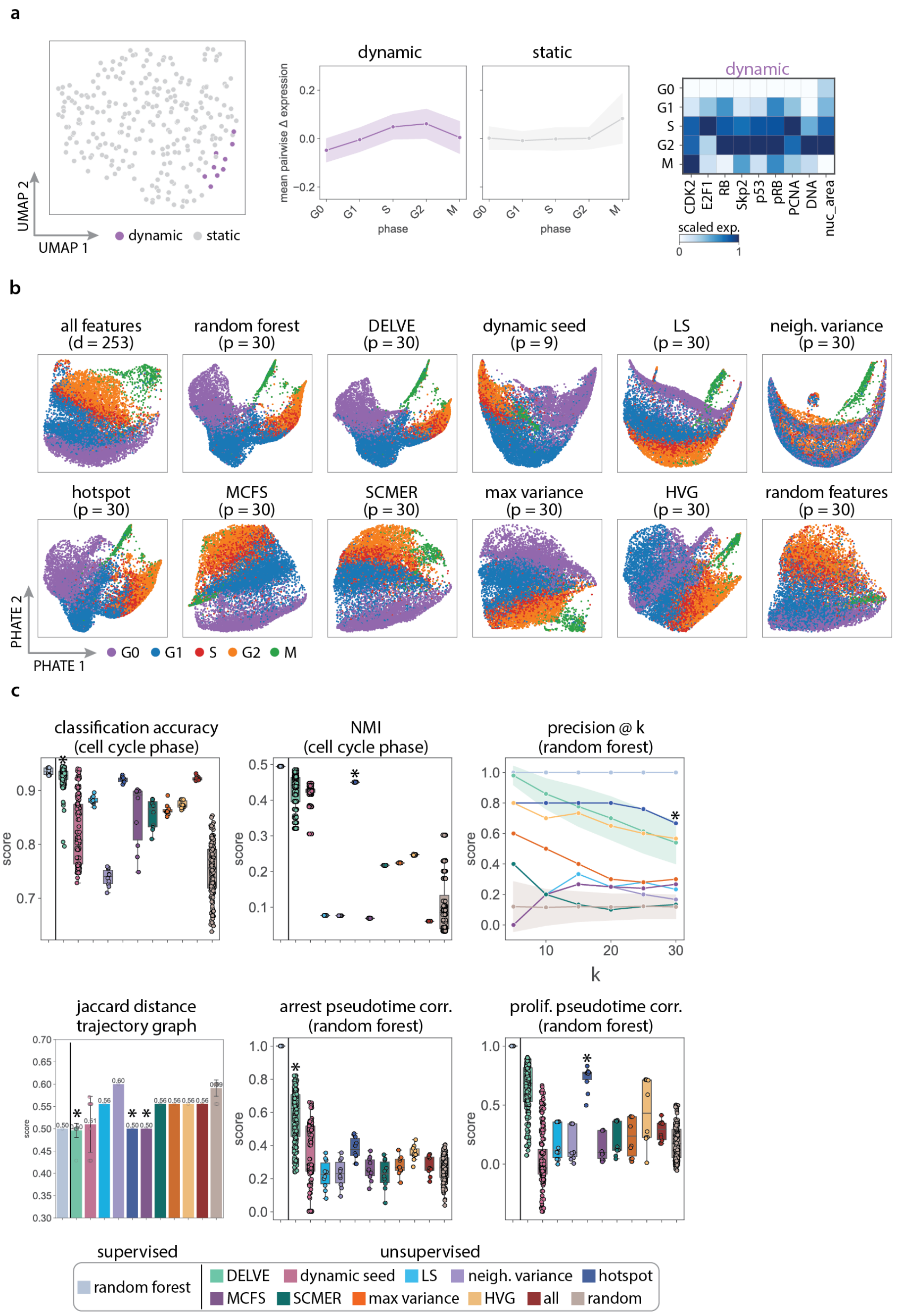

Supplementary Figure 25: DELVE recovers Pa16C pancreatic adenocarcinoma cell cycle trajectories in protein immunofluorescence imaging data. Pa16C cells were profiled with protein immunofluorescence imaging to measure 63 core cell cycle effectors resulting in a dataset with $d = 253$ imaging-derived features. (a) DELVE identified one module of dynamic features representing a minimum cell cycle. (a left) UMAP visualization of image-derived features where each point indicates a dynamic or static feature identified by the model. (a middle) The average pairwise change in expression for features within a module ordered across ground truth cell cycle phase annotations. (a right) Heatmap illustrating the standardized average expression of dynamic seed features across cell cycle phases. (b) Feature selection was performed to select the top $p = 30$ ranked features. Example PHATE visualizations of cell cycle trajectories for twelve feature selection approaches. (c) Quantitative assessment of twelve feature selection methods on preserving cell cycle phases and phase transitions according to several metrics including: support vector machine classification accuracy to the ground truth phase annotations, normalized mutual information (NMI) clustering score to ground truth phase annotations, precision of cell cycle phase-specific imaging-derived features as measured by a random forest classifier trained on ground truth phase annotations, Jaccard distance between predicted cell cycle trajectory graphs and a ground truth reference cell cycle trajectory curated from the literature, and the Kendall rank correlation between estimated pseudotime and the ground truth as measured by a random forest classifier trained on ground truth phase annotations. All error bands represent the standard deviation. All boxplots show the median (middle line), the interquartile range (upper and lower bounds of the box), and the minimum and maximum of the distributions (whiskers) over $n = 10$ random splits, seeds, or root cells. DELVE, dynamic seed, and random feature selection were run over $n = 20$ random trials to show reproducibility of the approach. * indicates the method with the highest median score. Source data are provided in a Source Data file.

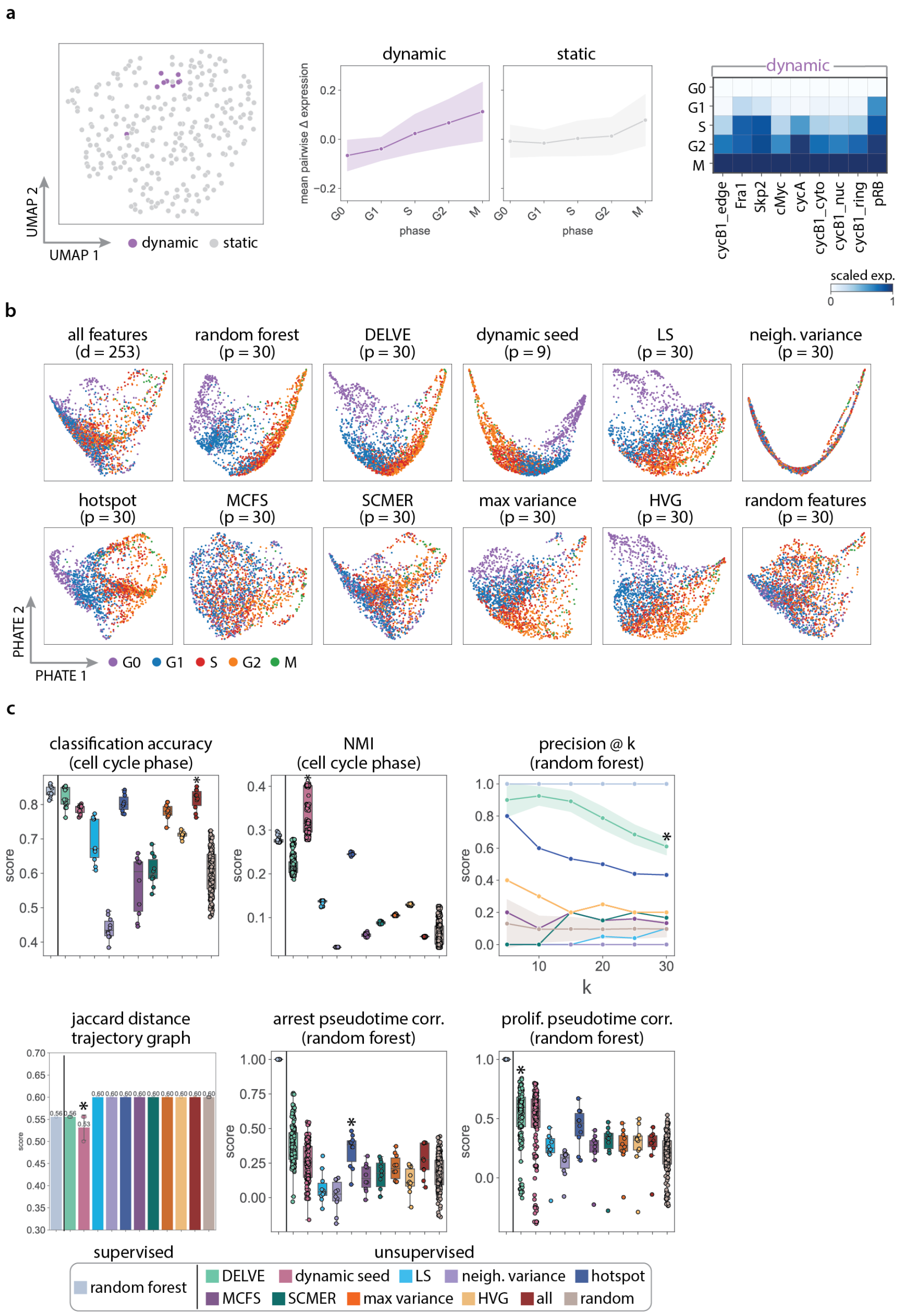

Supplementary Figure 26: DELVE recovers PANC1 pancreatic adenocarcinoma cell cycle trajectories in protein immunofluorescence imaging data. PANC1 cells were profiled with protein immunofluorescence imaging to measure 63 core cell cycle effectors resulting in a dataset with $d = 253$ imaging-derived features. (a) DELVE identified one module of dynamic features representing a minimum cell cycle. (a left) UMAP visualization of image-derived features where each point indicates a dynamic or static feature identified by the model. (a middle) The average pairwise change in expression for features within a module ordered across ground truth cell cycle phase annotations. (a right) Heatmap illustrating the standardized average expression of dynamic seed features across cell cycle phases. (b) Feature selection was performed to select the top $p = 30$ ranked features. Example PHATE visualizations of cell cycle trajectories for twelve feature selection approaches. (c) Quantitative assessment of twelve feature selection methods on preserving cell cycle phases and phase transitions according to several metrics including: support vector machine classification accuracy to the ground truth phase annotations, normalized mutual information (NMI) clustering score to ground truth phase annotations, precision of cell cycle phase-specific imaging-derived features as measured by a random forest classifier trained on ground truth phase annotations, Jaccard distance between predicted cell cycle trajectory graphs and a ground truth reference cell cycle trajectory curated from the literature, and the Kendall rank correlation between estimated pseudotime and the ground truth as measured by a random forest classifier trained on ground truth phase annotations. All error bands represent the standard deviation. All boxplots show the median (middle line), the interquartile range (upper and lower bounds of the box), and the minimum and maximum of the distributions (whiskers) over $n = 10$ random splits, seeds, or root cells. DELVE, dynamic seed, and random feature selection were run over $n = 20$ random trials to show reproducibility of the approach. * indicates the method with the highest median score. Source data are provided in a Source Data file.

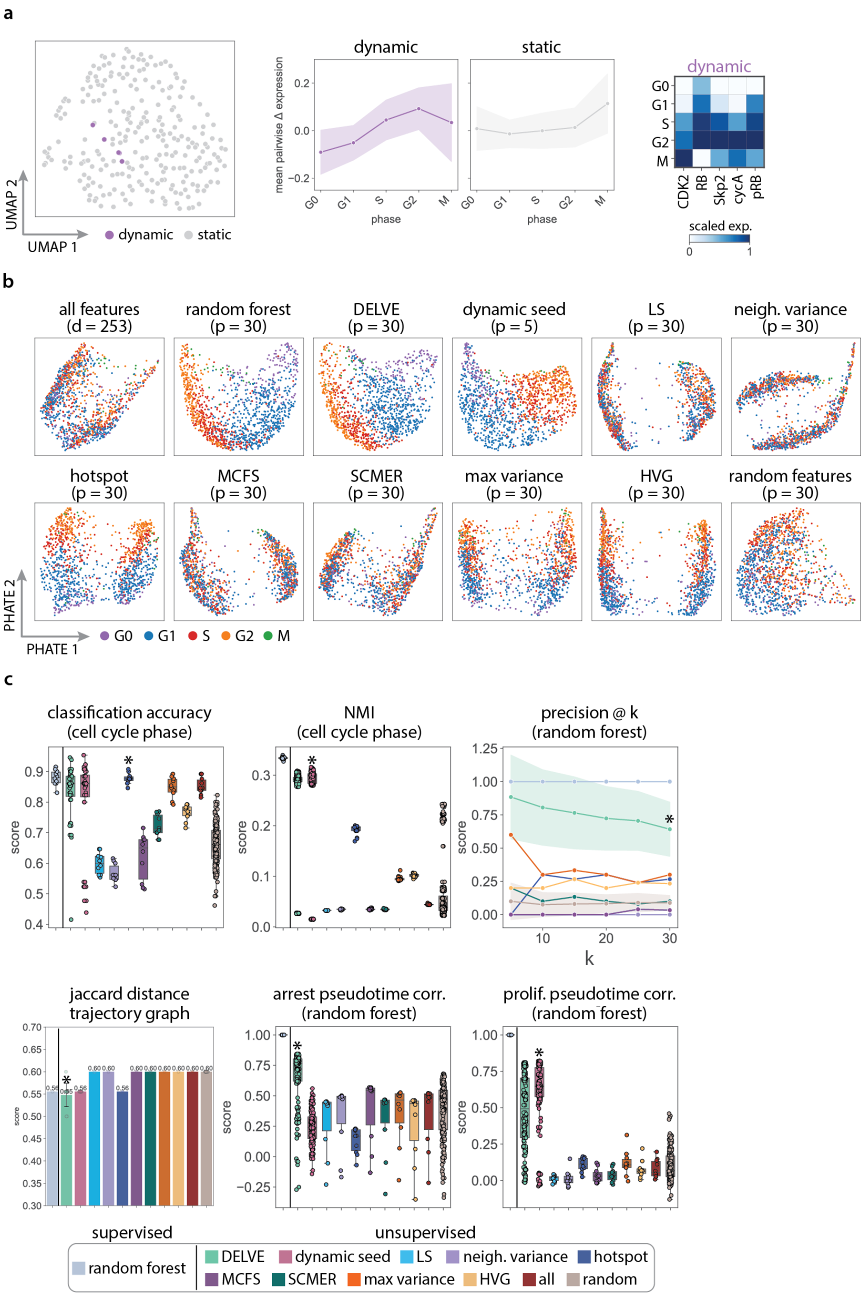

Supplementary Figure 27: DELVE recovers UM53 pancreatic adenocarcinoma cell cycle trajectories in protein immunofluorescence imaging data. UM53 cells were profiled with protein immunofluorescence imaging to measure 63 core cell cycle effectors resulting in a dataset with $d = 253$ imaging-derived features. (a) DELVE identified one module of dynamic features representing a minimum cell cycle. (a left) UMAP visualization of image-derived features where each point indicates a dynamic or static feature identified by the model. (a middle) The average pairwise change in expression for features within a module ordered across ground truth cell cycle phase annotations. (a right) Heatmap illustrating the standardized average expression of dynamic seed features across cell cycle phases. (b) Feature selection was performed to select the top $p = 30$ ranked features. Example PHATE visualizations of cell cycle trajectories for twelve feature selection approaches. (c) Quantitative assessment of twelve feature selection methods on preserving cell cycle phases and phase transitions according to several metrics including: support vector machine classification accuracy to the ground truth phase annotations, normalized mutual information (NMI) clustering score to ground truth phase annotations, precision of cell cycle phase-specific imaging-derived features as measured by a random forest classifier trained on ground truth phase annotations, Jaccard distance between predicted cell cycle trajectory graphs and a ground truth reference cell cycle trajectory curated from the literature, and the Kendall rank correlation between estimated pseudotime and the ground truth as measured by a random forest classifier trained on ground truth phase annotations. All error bands represent the standard deviation. All boxplots show the median (middle line), the interquartile range (upper and lower bounds of the box), and the minimum and maximum of the distributions (whiskers) over $n = 10$ random splits, seeds, or root cells. DELVE, dynamic seed, and random feature selection were run over $n = 20$ random trials to show reproducibility of the approach. * indicates the method with the highest median score. Source data are provided in a Source Data file.

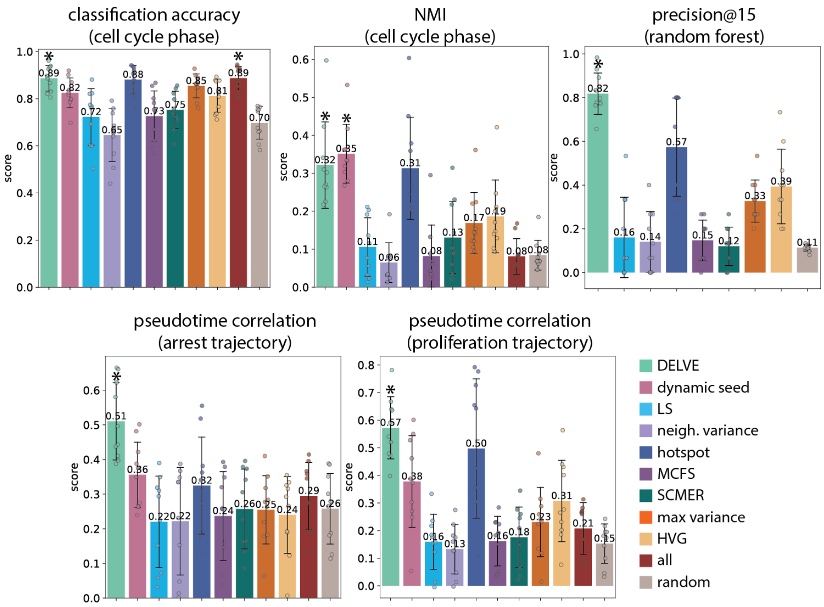

Supplementary Figure 28: DELVE outperforms existing feature selection methods on inferring cell cycle trajectories from protein immunofluorescence imaging data. Average performance of eleven unsupervised feature selection methods across ten indirect iterative immunofluoresence imaging (4i) datasets (RPE, BxPC3, CFPAC, HPAC, MiaPaCa, Pa02C, Pa01C, Pa16C, PANC1, UM53). For each dataset, feature selection was performed to select the top 30 cell cycle-specific features. Cell cycle preservation was then quantitatively assessed according to several metrics including: support vector machine classification accuracy to the ground truth phase annotations, normalized mutual information (NMI) clustering score to ground truth phase annotations, precision of cell cycle phase-specific imaging-derived features as measured by a random forest classifier trained on ground truth phase annotations, and the Kendall rank correlation between estimated pseudotime and the ground truth progression. The barplots represent the mean $\pm$ standard deviation across $n = 10$ proteomic imaging datasets. * indicates the method with the highest mean score. Source data are provided in a Source Data file.

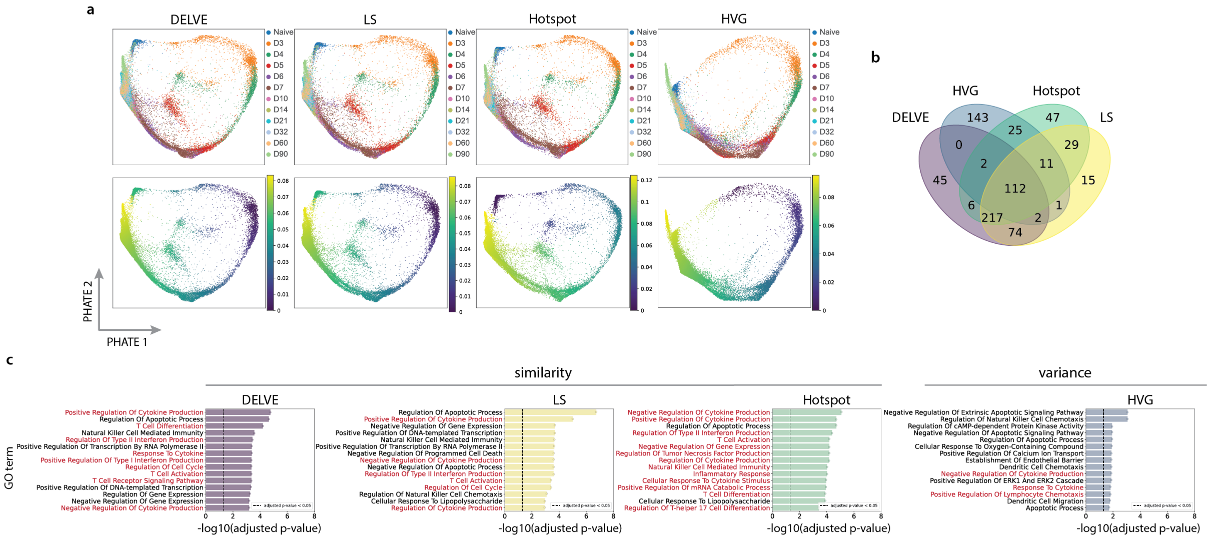

Supplementary Figure 29: Comparison of feature selection strategies on inferring CD8+ T cell differentiaton trajectories using Slingshot. (a) PHATE visualizations of the CD8+ T cell memory lineage for four feature selection strategies (DELVE, Laplacian score, Hotspot, highly variable gene (HVG) selection). Cells were colored according to (top) time following infection (bottom) estimated pseudotime using Slingshot. (b) Genes were regressed along estimated pseudotime using a generalized additive model to determine lineage-specific significant genes. The venn diagram illustrates the quantification and overlap of memory lineage-specific genes across feature selection strategies. (c) Barplots show the top 15 gene set enrichment terms associated with the temporally-expressed gene lists specific to each feature selection strategy following trajectory inference with Slingshot. p-values were computed using a Fisher exact test and adjusted with Benjamini Hochberg correction. Source data are provided in a Source Data file.

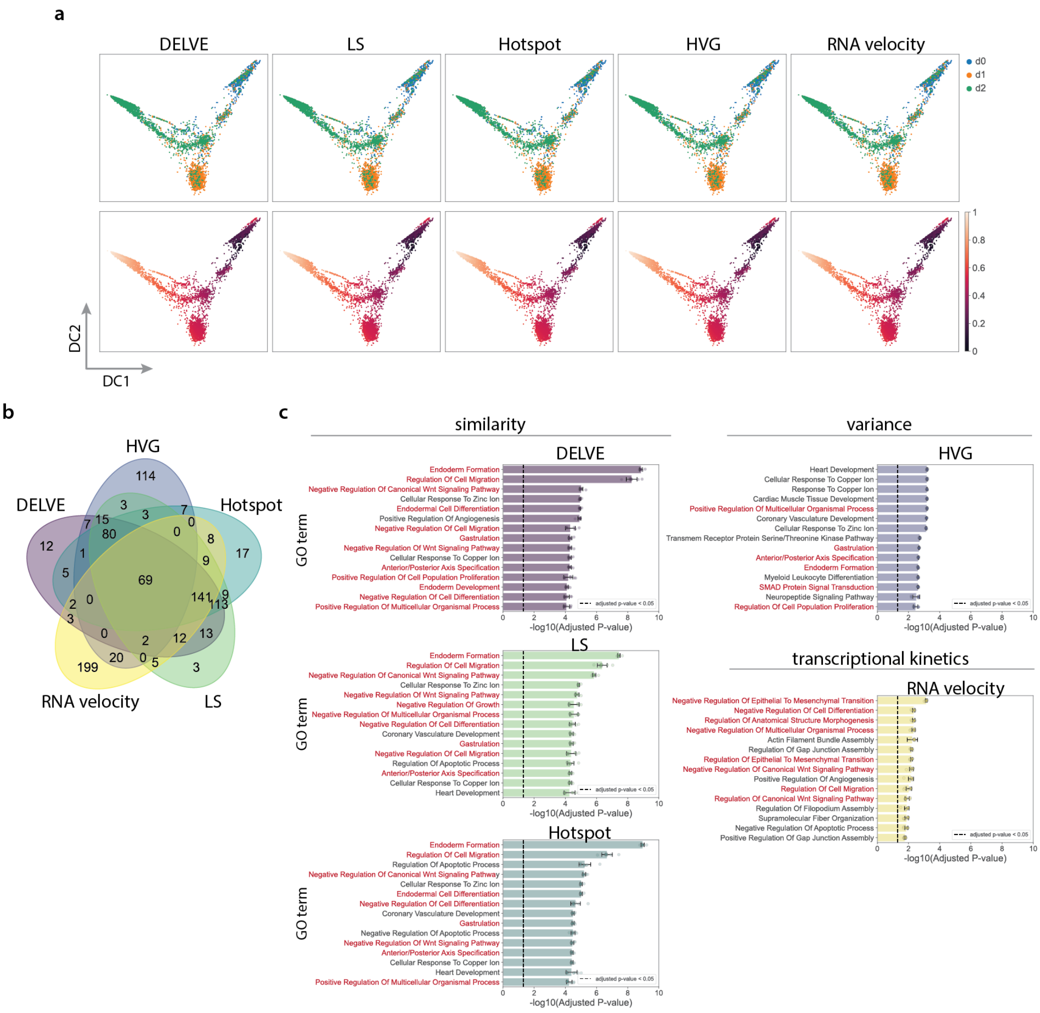

Supplementary Figure 30: Comparison of feature selection strategies on inferring definitive endoderm differentiation trajectories using diffusion pseudotime. (a) PHATE visualizations of the definitive endoderm lineage for four feature selection strategies (DELVE, Laplacian score, Hotspot, highly variable gene (HVG) selection). Cells were colored according to (top) time following induction with Activin A and CHIR99021 (bottom) estimated pseudotime using diffusion pseudotime. (b) Genes were regressed along estimated pseudotime using a generalized additive model to determine lineage-specific significant genes. The venn diagram illustrates the quantification and overlap of definitive endoderm lineage-specific genes across feature selection strategies. (c) Barplots show the mean $\pm$ standard deviation for the top 15 gene set enrichment terms associated with the temporally-expressed gene lists specific to each feature selection strategy following trajectory inference with $n = 10$ random root cells. p-values were computed using a Fisher exact test and adjusted with Benjamini Hochberg correction. Source data are provided in a Source Data file.

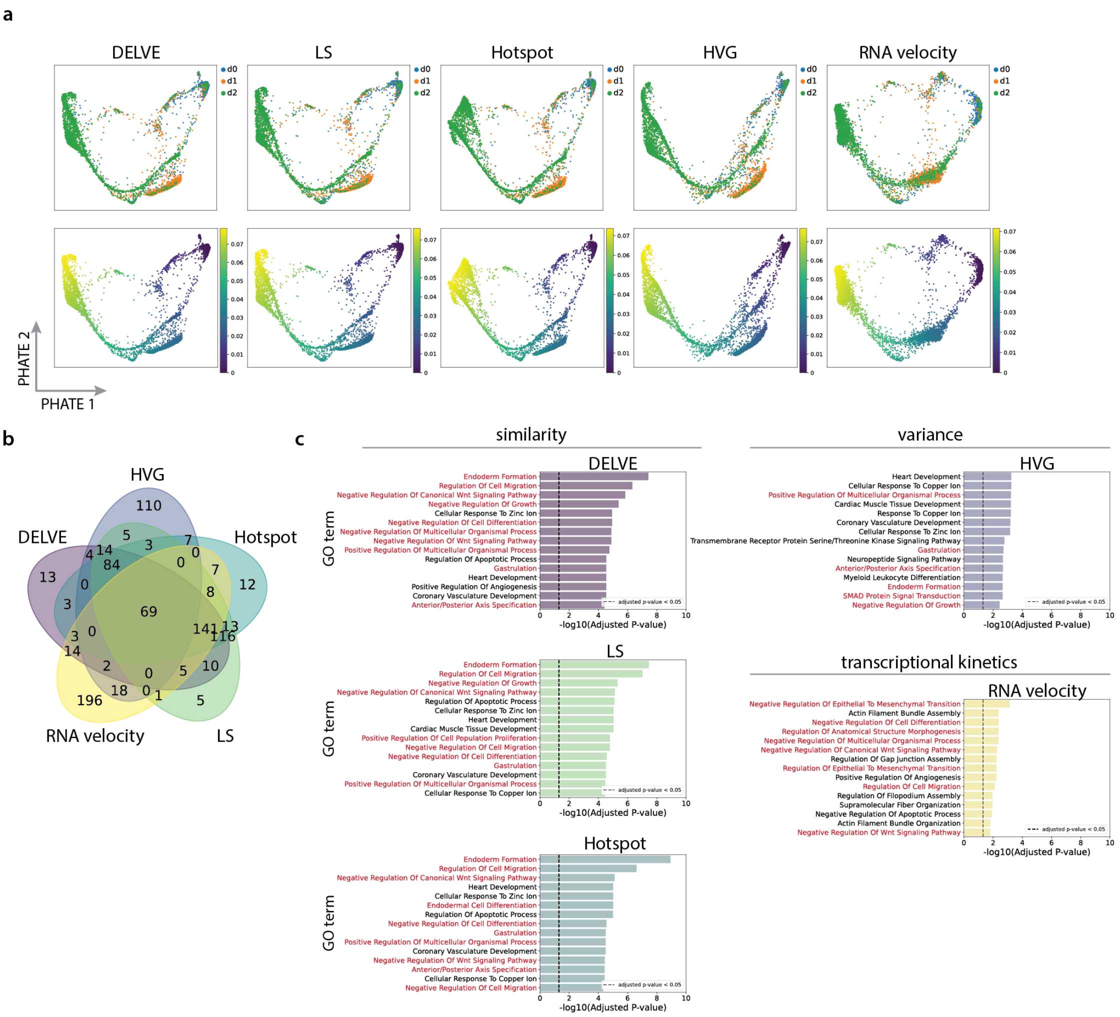

Supplementary Figure 31: Comparison of feature selection strategies on inferring definitive endoderm differentiation trajectories using Slingshot. (a) PHATE visualizations of the definitive endoderm lineage for four feature selection strategies (DELVE, Laplacian score, Hotspot, highly variable gene (HVG) selection). Cells were colored according to (top) time following induction with Activin A and CHIR99021 (bottom) estimated pseudotime using Slingshot. (b) Genes were regressed along estimated pseudotime using a generalized additive model to determine lineage-specific significant genes. The venn diagram illustrates the quantification and overlap of definitive endoderm lineage-specific genes across feature selection strategies. (c) Barplots show the top 15 gene set enrichment terms associated with the temporally-expressed gene lists specific to each feature selection strategy following trajectory inference with Slingshot. p-values were computed using a Fisher exact test and adjusted with Benjamini Hochberg correction. Source data are provided in a Source Data file.
