# Supplementary material for: DELVE: feature selection for preserving biological trajectories in single-cell data
Source: Nat Commun. 2024 Mar 29;15:2765. doi: 10.1038/s41467-024-46773-z (PMC10980758; doi:10.1038/s41467-024-46773-z)
Supplement: Supplementary file 3 — Reporting Summary [file 41467_2024_46773_MOESM3_ESM.pdf]

nature portfolio

Corresponding author(s): Natalie Stanley and Jeremy E. Purvis

Last updated by author(s): 1/19/2024

Reporting Summary

Nature Portfolio wishes to improve the reproducibility of the work that we publish. This form provides structure for consistency and transparency in reporting. For further information on Nature Portfolio policies, see our Editorial Policies and the Editorial Policy Checklist.

Statistics

For all statistical analyses, confirm that the following items are present in the figure legend, table legend, main text, or Methods section.

n/a	Confirmed	
☐	☒	The exact sample size (*n*) for each experimental group/condition, given as a discrete number and unit of measurement
☐	☒	A statement on whether measurements were taken from distinct samples or whether the same sample was measured repeatedly
☐	☒	The statistical test(s) used AND whether they are one- or two-sided<br>*Only common tests should be described solely by name; describe more complex techniques in the Methods section.*
☒	☐	A description of all covariates tested
☐	☒	A description of any assumptions or corrections, such as tests of normality and adjustment for multiple comparisons
☐	☒	A full description of the statistical parameters including central tendency (e.g. means) or other basic estimates (e.g. regression coefficient) AND variation (e.g. standard deviation) or associated estimates of uncertainty (e.g. confidence intervals)
☐	☒	For null hypothesis testing, the test statistic (e.g. *F*, *t*, *r*) with confidence intervals, effect sizes, degrees of freedom and *P* value noted<br>*Give P values as exact values whenever suitable.*
☒	☐	For Bayesian analysis, information on the choice of priors and Markov chain Monte Carlo settings
☒	☐	For hierarchical and complex designs, identification of the appropriate level for tests and full reporting of outcomes
☒	☐	Estimates of effect sizes (e.g. Cohen's *d*, Pearson's *r*), indicating how they were calculated

Our web collection on statistics for biologists contains articles on many of the points above.

Software and code

Policy information about availability of computer code

Data collection	The scripts to download and preprocess the single-cell datasets or generate the simulated datasets are available in the delve_benchmark GitHub repository: https://github.com/jranek/delve_benchmark and in the Zenodo repository: https://zenodo.org/records/10426508. All d Splatter simulated datasets were generated using the scprep v1.2.3 SplatSimulate wrapper function in Python >= 3.6 using Splatter v1.18.2 in R v4.1.1. SymSim simulated datasets were generated using SymSim v0.0.0.9000 in R v4.1.1. Moreover, all software used in this manuscript are detailed in the venv_delve_benchmark.yml file within the GitHub repository.
Data analysis	hotspotsc v1.1.1, scmer v0.1.0a3, gseapy v1.0.4, scikit-learn v0.23.2, statsmodels v0.14.0, phate v1.0,11, umap-learn v0.5.1, scanpy v1.9.3, scprep v1.2.3, networkx v3.1 packages in python >= v3.6 . Splatter v1.18.2, symsim v0.0.0.9000, slingshot v2.1.1 packages in R v4.1.1, and several custom functions within our GitHub repository (https://github.com/jranek/delve_benchmark) were used to analyze the data.

For manuscripts utilizing custom algorithms or software that are central to the research but not yet described in published literature, software must be made available to editors and reviewers. We strongly encourage code deposition in a community repository (e.g. GitHub). See the Nature Portfolio guidelines for submitting code & software for further information.

Data

Policy information about availability of data

All manuscripts must include a data availability statement. This statement should provide the following information, where applicable:

- Accession codes, unique identifiers, or web links for publicly available datasets
- A description of any restrictions on data availability
- For clinical datasets or third party data, please ensure that the statement adheres to our policy

The raw publicly available single-cell datasets used in this study are available in the Zenodo repository (https://doi.org/10.5281/zenodo.4525425) for the RPE cell cycle dataset, the Zenodo repository (https://doi.org/10.5281/zenodo.7860332) for the PDAC cell cycle datasets, and the Gene Expression Omnibus (GEO) under the accession code GSE131847 for the CD8+ T cell differentiation dataset. All preprocessed datasets, including the DE differentiation dataset are available in the Zenodo repository (https://doi.org/10.5281/zenodo.10534873). The STRING database leveraged in this study is available at (https://string-db.org/). The source data are provided with this paper and available in the Zenodo repository (https://doi.org/10.5281/zenodo.10534873). There are no restrictions on data availability.

Research involving human participants, their data, or biological material

Policy information about studies with human participants or human data. See also policy information about sex, gender (identity/presentation), and sexual orientation and race, ethnicity and racism.

Reporting on sex and gender	N/A
Reporting on race, ethnicity, or other socially relevant groupings	N/A
Population characteristics	N/A
Recruitment	N/A
Ethics oversight	N/A

Note that full information on the approval of the study protocol must also be provided in the manuscript.

Field-specific reporting

Please select the one below that is the best fit for your research. If you are not sure, read the appropriate sections before making your selection.

[x] Life sciences   [ ] Behavioural & social sciences   [ ] Ecological, evolutionary & environmental sciences

For a reference copy of the document with all sections, see nature.com/documents/nr-reporting-summary-flat.pdf

Life sciences study design

All studies must disclose on these points even when the disclosure is negative.

Sample size	For the publicly available iterative indirect immunofluoresence imaging data and single-cell RNA sequencing data, sample sizes were determined by the authors of the original study. No sample size calculations were performed for the RPE (PMID 34800361), PDAC (https://doi.org/10.5281/zenodo.7860332), CD8 (PMID 32414833), or DE datasets.
Data exclusions	Data exclusions were determined according to (1) quality control measures such as the distribution of molecular counts and expression of mitochondrial markers, and/or (2) the availability of ground truth cellular annotations. These determinations are fully described in the Methods section of the manuscript.
Replication	For the publicly available data used in this study, the number of replicates were determined by the authors of the original study. The RPE study collected data in technical duplicates (PMID 34800361), the PDAC study had a single well per cell line (https://doi.org/10.5281/zenodo.7860332), and the CD8T cell differentiation study collected CD8T cells that were pooled from approximately 1-6 mice at each timepoint. Here each timepoint of the analysis represented an independent experiment (PMID 32414833). For the DE differentiation dataset, cells were randomly allocated into three replicate wells per treatment condition and pooled prior to acquisition. The DE differentiation study was performed twice independently and successful.
Randomization	For the publicly available data used in this study, the randomization was determined by the authors of the original study. The RPE (PMID 34800361) and PDAC (https://doi.org/10.5281/zenodo.7860332) studies had no randomization as there was no treatment induction for the control data. For the CD8T cell study, mice were randomly allocated into groups before adoptive transfer and mice were randomly selected for cell harvesting at specific time points (PMID 32414833). For the DE dataset, cells were randomly placed into three replicate wells for each condition, treated with differentiation stimuli, and pooled prior to acquisition.
Blinding	There was no blinding in this study as it was not relevant for analysis of feature selection method performance.

Reporting for specific materials, systems and methods

We require information from authors about some types of materials, experimental systems and methods used in many studies. Here, indicate whether each material, system or method listed is relevant to your study. If you are not sure if a list item applies to your research, read the appropriate section before selecting a response.

Materials & experimental systems

n/a	Involved in the study	
☒	☐	Antibodies
☐	☒	Eukaryotic cell lines
☒	☐	Palaeontology and archaeology
☒	☐	Animals and other organisms
☒	☐	Clinical data
☒	☐	Dual use research of concern
☒	☐	Plants

Methods

n/a	Involved in the study	
☒	☐	ChIP-seq
☒	☐	Flow cytometry
☒	☐	MRI-based neuroimaging

Eukaryotic cell lines

Policy information about cell lines and Sex and Gender in Research

Cell line source(s)	H9 human embryonic stem cells (WA09) were obtained from WiCell.
Authentication	H9 human embryonic stem cell line are a NIH approved cell line that has been authenticated and stored under GMP conditions from WiCell. The Human Pluripotent Cell Core at UNC periodically performs STR profiling to confirm cell line identity. This is done as a fee-for service through commercial vendors (IDEXX Bioresearch). The core routinely screens for consistency of morphological characteristics and generally passage cultures for 8-10 weeks before starting them fresh from frozen stocks. They test for mycoplasma contamination routinely using Hoechst staining and commercially available PCR tests. Infected cells are discarded, and new vials of fresh cells are thawed for use.
Mycoplasma contamination	The H9 human embryonic stem cell line was tested negative for mycoplasma from the Human Pluripotent Cell Core at UNC.
Commonly misidentified lines (See ICLAC register)	No commonly misidentified cell line was used in this study.

Plants

Seed stocks	N/A
Novel plant genotypes	N/A
Authentication	N/A
